# Nucleation-Limited Kinetics of GaAs Nanostructures Grown by Selective Area Epitaxy: Implications for Shape Engineering in Optoelectronics Devices

**DOI:** 10.1021/acsanm.4c02765

**Published:** 2024-08-13

**Authors:** Michele Zendrini, Vladimir Dubrovskii, Alok Rudra, Didem Dede, Anna Fontcuberta i Morral, Valerio Piazza

**Affiliations:** † Laboratory of Semiconductor Materials, Institute of Materials, Ecole Polytechnique Fédérale de Lausanne (EPFL), Lausanne CH-1015, Switzerland; ‡ Faculty of Physics, St. Petersburg State University, Universitetskaya Emb. 13B, St. Petersburg 199034, Russia; § Institute of Physics, Ecole Polytechnique Fédérale de Lausanne (EPFL), Lausanne CH-1015, Switzerland

**Keywords:** SAE, MOVPE, GaAs, nanowires, growth kinetics

## Abstract

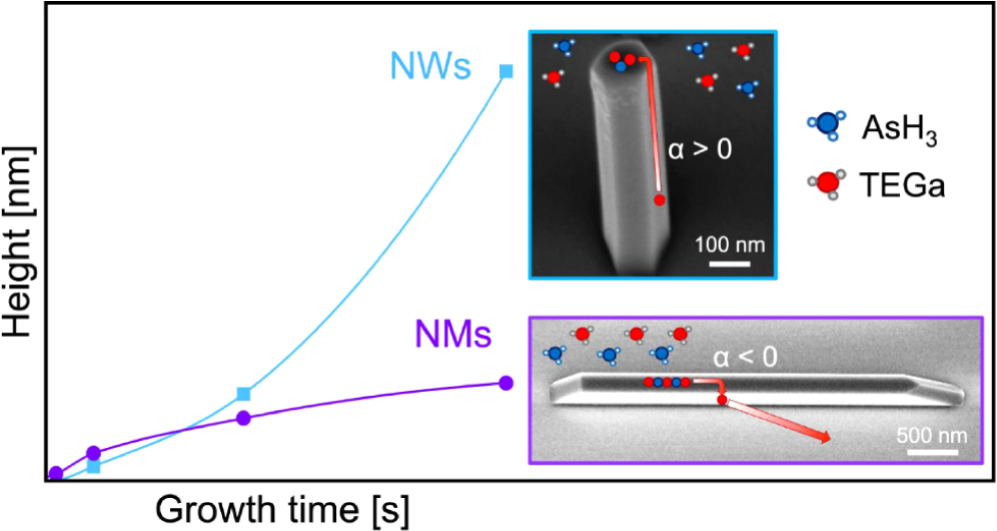

The growth kinetics of vertical III–V nanowires (NWs) were clarified long ago. The increasing aspect ratio of NWs results in an increase in the surface area, which, in turn, enhances the material collection. The group III adatom diffusion from the NW sidewalls to the top sustains a superlinear growth regime. In this work, we report on the growth of different GaAs nanostructures by selective area MOVPE on GaAs (111)B substrates. We show that the opening dimensions and geometry qualitatively alter the morphology and height evolution of the structures. We compare the time evolution of vertical GaAs NWs stemming from circular holes and horizontal GaAs nanomembranes (NMs) growing from one-dimensional (1D) rectangular slits on the same substrate. While NW heights grow exponentially with time, NMs surprisingly exhibit sublinear kinetics. The absence of visible atomic steps on the top facets of both NWs and NMs suggests layer-by-layer growth in the mononuclear mode. We interpret these observations within a self-consistent growth model, which links the diffusion flux of Ga adatoms to the position- and shape-dependent nucleation rate on top of NWs and NMs. Specifically, the island nucleation rate is lower on top of the NMs than that on the NWs, resulting in the total diffusion flux being directed from the top facet to the sidewalls. This gives a sublinear height evolution for the NMs. These results open innovative perspectives for shape engineering of III–V nanostructures and new avenues for the design of optoelectronics and photonic devices.

## Introduction

Selective area epitaxy (SAE) enables the fabrication of III–V nanostructures with a high degree of control over their position, dimensions, and shapes.^1,2^ The SAE approach allows for the formation of uniform arrays of vertical nanowires (NWs), their horizontal counterparts, and networks, which are used in solar cells, microlasers, or THz detectors.^3−5^ SAE-grown vertical NWs are promising platforms for optoelectronics because of their unique light absorption enhancement, polarization sensitivity, and optical mode waveguiding.^6−8^ Difficult scalability over large areas and complex device processing of free-standing NWs have recently pushed research toward horizontal nanomembranes (NMs), whose integration into functional devices is highly compatible with widespread manufacturing processes. Free-standing NWs have been studied for a much longer time, resulting in a stronger understanding and control over their growth and properties.^8^ It has been demonstrated that the mechanisms ruling the crystal structure largely influence the functionality of both NWs and NMs.^9−11^ Thorough investigations of the growth mechanisms laid the base for the engineering of the morphology and crystal phase of these nanostructures.^12−16^ This work aims to clarify key aspects of the nanoscale phenomena that govern the growth of both NWs and NMs, thus extending tailoring and control of growth parameters.

Demonstrations of position-controlled growth of high aspect-ratio GaAs NWs with predefined diameters via the gold catalyzed vapor–liquid–solid (VLS), self-catalyzed, or vapor–solid (VS) mechanisms date back to the beginning of the 2000s.^17−20^ Since then, our comprehension of the growth mechanisms has increased to a sophisticated level.^21−24^ This progress has led to the achievement of precise control on nanowire dimensions, orientation, and morphology.^25−29^ Recently, the capability of observing nanowire growth at the atomic scale in an in situ transmission electron microscope (TEM) has further increased the comprehension of nanowire growth and opened new questions.^30,31^ In particular, several groups have demonstrated the role of the catalyst contact angle in polytypism in different material systems.^32−35^ For example, in self-catalyzed growth of GaAs NWs, the contact angle of the Ga droplet determines if the growth interface is planar or truncated. In turn, this results in a different crystal phase: cubic zincblende (ZB) at small (<100°) and large (>125°) contact angles, and hexagonal wurtzite (WZ) at intermediate contact angles.^13^ It is now well established that the VLS growth of III–V NWs can be limited either by the material transport of group III and V species, including surface diffusion of group III adatoms,^36−42^ or by the nucleation of two-dimensional (2D) islands on the NW top facet in the mononuclear regime.^43−47^ Self-consistent modeling linking the material transport mechanism with 2D nucleation is rare even for the most studied VLS III–V NWs.^40,42,43,46^ The exponential time dependence of the NW height is commonly observed in VLS NWs and attributed to the surface diffusion of group III adatoms from the NW sidewalls to their top facets.^36−38,40,41^ Negative, or downward, diffusion from the NW tops to their sidewalls was considered in connection with the NW dissolution in the absence of group III flux,^36^ but never observed for NWs growing under standard conditions. Consequently, the relationship between the direction of the surface diffusion flux and 2D nucleation on the top facet still needs to be studied. However, it is well-known that the nucleation often occurs at very specific crystal sites.^48,49^ The in situ growth monitoring of self-catalyzed VLS GaAs NWs demonstrates that the monolayer progression advances by atomic step flow starting at the corners of the top facet of hexahedral NWs.^34^

Furthermore, surface diffusion of group III adatoms plays a crucial role in the growth dynamics of both VLS and VS grown III–V NWs. Systematic studies of the morphological evolution of VS GaAs^50^ and InAs^51^ NWs as a function of the opening size and spacing showed a superlinear increase in the NW height and volume with time, similar to VLS NWs.^36−38,40,41^ Without a catalyst, the adatom diffusion over a given length of the NW controls the VS growth.^52^ These findings suggest that the VS growth kinetics of III–V NW under group V-rich conditions are naturally diffusion-controlled, independent of the top facet geometry. However, in all previous studies, nucleation-related limitations of the VS NW growth were, to the best of our knowledge, never considered.

NMs are out-of-plane extended versions of horizontal NWs. These structures stem from rectangular openings (slits) characterized by their width and length, different from the holes commonly used for NWs where the size is defined by their diameter. Under group V-rich conditions, NMs grow to controllable heights. The impact of pattern feature sizes and growth parameters on the final morphology depends on the direction of the slits and the crystal orientation of the substrate.^53−56^ The growth rate of NMs highly depends on the width and the slit-to-slit distance (hereon pitch) when grown by molecular beam epitaxy (MBE).^57^ The spacing between features has a relevant impact due to the cooperative role of incoming fluxes, diffusion processes, and desorption mechanisms that determine the final growth mode of NMs.^58^ Differently, the pitch dependence is less significant for MOVPE-grown NMs due to the long diffusion lengths of precursors.^59^ On the whole, it is particularly important to clarify the mechanisms in the creation of the NMs as the faceting of these nanostructures is kinetically driven, as demonstrated by Albani et al.^60,61^ In general, the kinetic models of this system rely on diffusion-limited conditions, as in the case of NWs, without considering 2D nucleation on the NM top facet as a possible limiting step for SAE growth.^62^

In this work, we compare the growth mechanisms of GaAs NMs and NWs by selective MOVPE on GaAs (111)B substrates. Both NWs and NMs are grown simultaneously on the same substrate, and the growth conditions are fixed and identical. We find that symmetrical hexahedral NWs with vertical (110) side facets exhibit the expected superlinear (exponential) increase of height, while the height evolution of asymmetrical and tapered NMs is sublinear. Similar to GaAs NMs grown on GaAs (100) substrates,^59^ the evolution of the top facet of the NMs suggests a layer-by-layer growth. To explain such differences, we propose a self-consistent model that takes into account the facet/surface dependent adatom diffusion processes and the shape-dependent nucleation at the top of the nanostructures. We show that the nucleation rate of tapered islands on top of NMs is much slower than that of islands with vertical facets on top of NWs. The insufficient nucleation rate on top of NMs redirects the surface diffusion of Ga adatoms from upward to downward, thus explaining the striking difference in the growth evolution. To the best of our knowledge, this is the first work where simultaneous SAE of III–V nanostructures of different dimensionality along with the nucleation-dependent direction of surface diffusion fluxes is studied. These findings open a new path to the morphological design of nanocrystals with complex shapes, which is the key for a range of electronic and photonic devices and has general, far-reaching implications in crystal growth.

## Experimental Results

**Figure 1:**
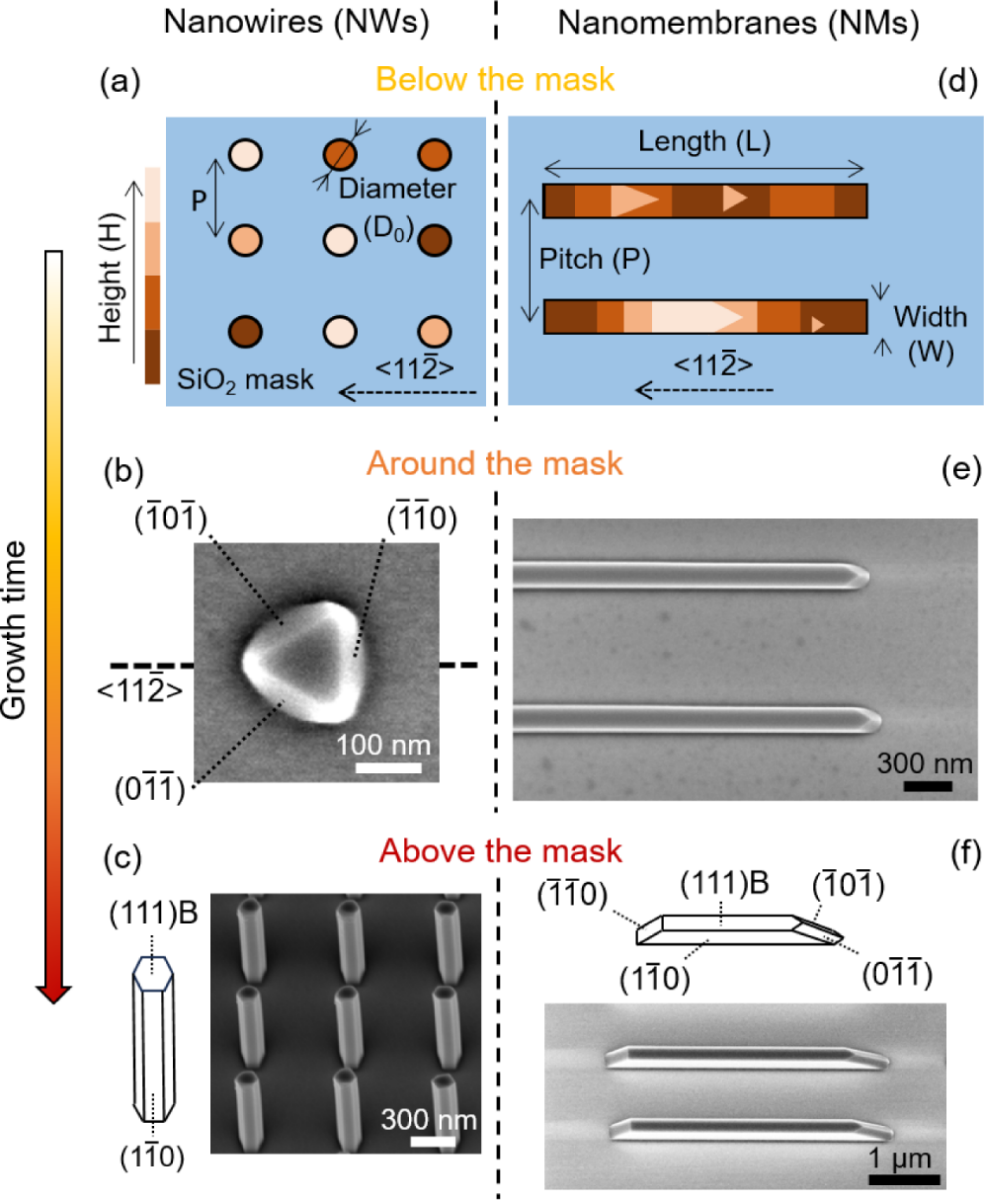
Overview of the morphological evolution of GaAs NWs and NMs. Below the mask: schematics of the patterns in the SiO_2_ mask. The color scale indicates the growth profiles of GaAs in (a) circular holes and (d) elongated slits in the early stages. Around the mask: SEM images of (b) NWs and (e) NMs with heights comparable to the mask thickness. The NMs exhibit stable facets after 180 s, while NWs have an incomplete triangular shape at 120 s. Above the mask: SEM images of the fully formed (c) hexahedral NWs and (f) tapered NMs with corresponding schematics of the facet orientation. Both nanostructures exhibit only (1–10) lateral facets.

GaAs nanostructures are grown by selective area MOVPE on GaAs (111)B substrates. The substrate surface is covered with a 25 nm SiO_2_ mask, where a growth pattern is defined by electron beam lithography and transferred by dry etching. The substrate fabrication is described in detail in the Methods section. We pattern circular holes and elongated slits along the <11> direction on the substrates to enable SAE of vertical NWs and horizontal NMs, respectively. The holes have nominal diameters *D*_0_ of 80, 112, 138, and 160 nm, and they are arranged in a square array with a fixed pitch (*P*) of 750 nm. The slits are 20 μm long. Each array has a different nominal width of the slits *W*_0_ and pitch *P*, which represents the distance between the centers of the adjacent slits. We investigate GaAs NMs grown in the arrays with nominal widths of 40, 80, and 140 nm and pitches of 500, 1000, 2000, and 4000 nm. Figure 1a–d illustrates the geometries of the pinhole and slit arrays.

After the substrate fabrication, thermal annealing is performed in the MOVPE reactor at 850 °C set temperature and under arsine flow for 4 min to remove the native oxide and promote nucleation in the openings. After this step, slits and holes have an average depth of 19.2 ± 6.5 and 19.3 ± 1.5 nm, respectively (see Figure S1). We use triethylgallium (TEGa) and AsH_3_ as precursors for GaAs growth at 800 °C under a V/III flux ratio of 1. GaAs NMs and NWs are grown simultaneously for various lengths of time, ranging from 30 to 840 s. We investigate the morphology of the grown samples by scanning electron microscopy (SEM) and atomic force microscopy (AFM), which allow us to fully evaluate the evolution of both the lateral and vertical dimensions of the structures.

Figure 1 summarizes the temporal shape evolution of GaAs NWs (Figure 1a,c) and NMs (Figure 1d,f) with time. We divided the growth into three steps depending on the grown volume with respect to the mask level. Figure 1a–d illustrates the geometries of the pinhole and slit arrays and demonstrates the early stages of growth. The representative AFM data are given in Figure S2. Figure 1b shows the truncated NWs when they grow to a height comparable to the mask level. These GaAs crystals in holes exhibit a triangular prism formed by the top (111)B facet and the lateral facets belonging to the {110} family. These 3-fold symmetrical structures form, twin, and pile up until hexahedral NWs grow outside the mask, as in refs^18^ and^19^. Figure 1c shows the schematic representation and a tilted SEM image of the fully grown NWs. This morphological evolution of GaAs NWs in SAE is well-known and extensively reported in the literature.^18−20,63^

Figure 1d depicts GaAs NMs within the slits when they are still growing below the mask level. These incomplete NMs display triangular islands with clearly visible multiple steps separating the terraces. Figure 1e shows an SEM image of the NMs array after growing out of the slits. These structures rapidly reach a stable shape and faceting. Figure 1f exhibits a tilted SEM image of the fully grown NMs and the corresponding schematics describing the facets. There are different factors driving the final shape, which are studied in refs^57^ and^58^. Importantly, NMs do not reproduce the rectangular shape of the slits, which would require the formation of vertical (211) sidewalls on the short sides. Instead, the NMs exhibit tapered shapes with a low index {110} family of facets on the short edges; one of which is flat and the other has the shape of a ridge inclined with respect to the vertical. We discuss this energetically preferred shape in the modeling part. Based on Figure 1, we can infer that the stable crystallographic configuration of both NWs and NMs contains only the (110) side facets and the flat (111B) top facet. Due to the difference in the template geometry, the lateral facets on the short sides of NMs must be inclined. The stability of the flat (111)B top of the NMs is also interesting. Previous works on MBE growths of GaAs NMs on GaAs (111)B substrates report top facets belonging to the (113) family.^60^ Nonetheless, the presence of a top (111)B facet is not surprising as the same difference in the facet orientation is observed between MBE- and MOVPE-grown GaAs NMs on (100) substrates.^58,59^ These growth modes lead to principally different shapes in the large time limit. Prior works reported the full completion of the structures, until the connection of the extending inclined facets, thus forming triangular slabs.^57^ In our growth conditions, inclined facets do not extend fast enough, thus always leaving a flat (111)B top facet (Figure S4).

**Figure 2:**
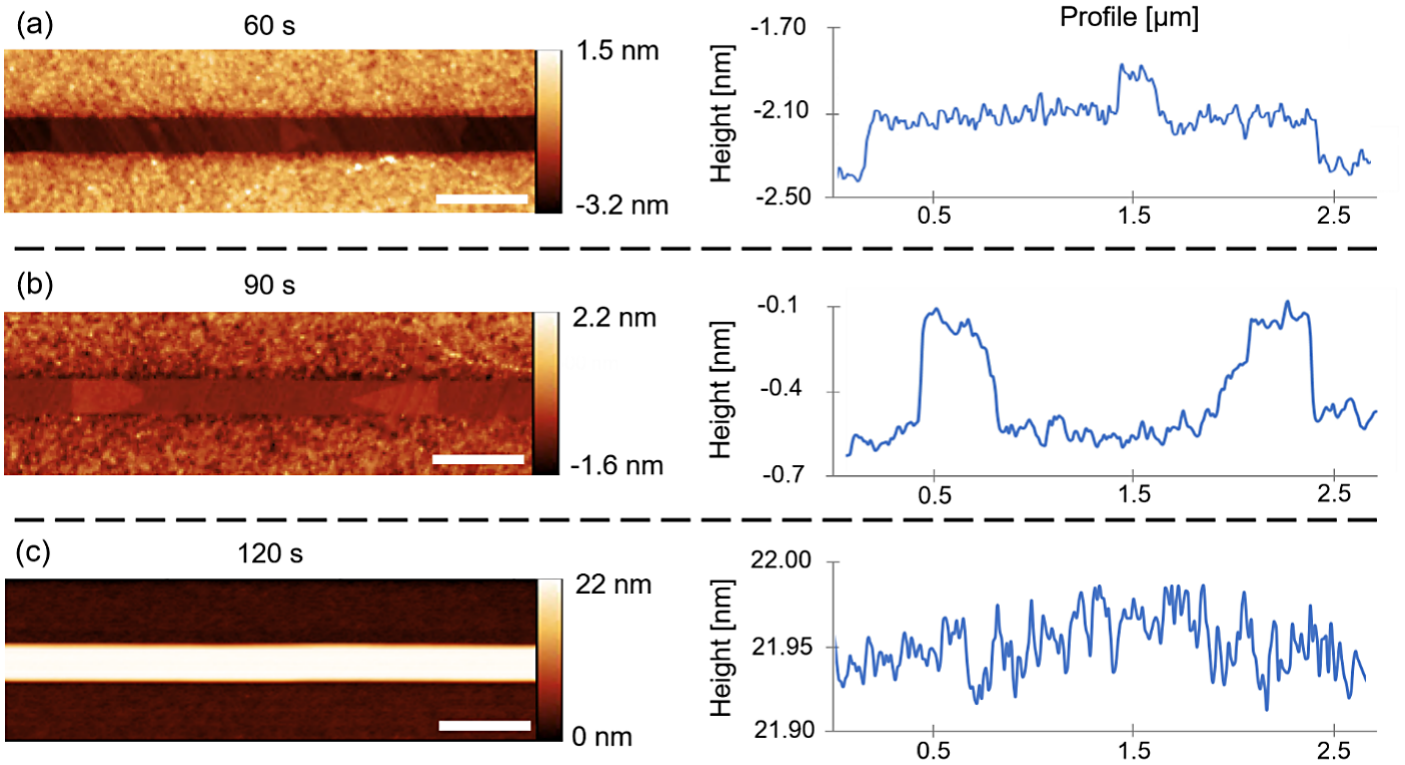
Island and terraces in the early stages of NM growth. AFM images of the slits after 60 (a) and 90 s (b) of growth, evidencing triangular islands and terraces, and corresponding line profile demonstrating monolayer thickness. (c) AFM image of the slits after 120 s of growth and the corresponding line profile, showing the absence of atomic steps once the NMs grow outside the mask. Scale bars correspond to 500 nm. Line profiles are taken in the middle of the slit and averaged on three points in width.

We now turn to analysis of the surface morphology of the NM top facets at different growth times. Figure 2a,b shows atomically flat terraces and islands formed inside the slits. The corresponding AFM line profiles clearly show the monolayer nature of both islands and terraces, with a ∼0.3 nm step height.^64^ Triangular islands have edges parallel to the {0} family. These islands appear to have the same crystallographic features of the lateral facets as the triangular NW seeds observed in the early growth stages,^18,19^ showed in Figure 1b–d. The islands in the early stages of the NMs growth have sizes up to almost half a micron. They appear to turn into larger terraces after merging and expand over several micrometers within the slits. These monolayer terraces manifest layer-by-layer growth within the mask, consistently with the similar structures grown by MOVPE on GaAs (100) substrates.^59^ When the NMs reach the height of the mask, such terraces remain uniquely visible at the edges of the slits (Figure S2). The growth front is no longer observed in NMs protruding from the mask. This indicates an extremely rapid longitudinal propagation of the steps along the entire NM length. Figure 2c shows the top facet of the NMs growing out of the mask for just a few nanometers and their corresponding AFM line profile. In this case, no step is observable over several microns, and the whole top facet appears atomically flat. The same is observed for longer growth times (see Figure S3 for further details). These data suggest that the propagation of atomic steps is faster for NMs outside of the mask. In this growth stage, we observe the typical mononuclear growth regime,^45,46^ where the vertical growth rate is determined by the vacant time between the successive nucleation events on the top facet rather than the time required for a monolayer island to fill the whole top facet once nucleated. According to the data, the mononuclear growth mode is observed in developed 20 μm long GaAs NMs, without any evidence of polynucleation.

**Figure 3:**
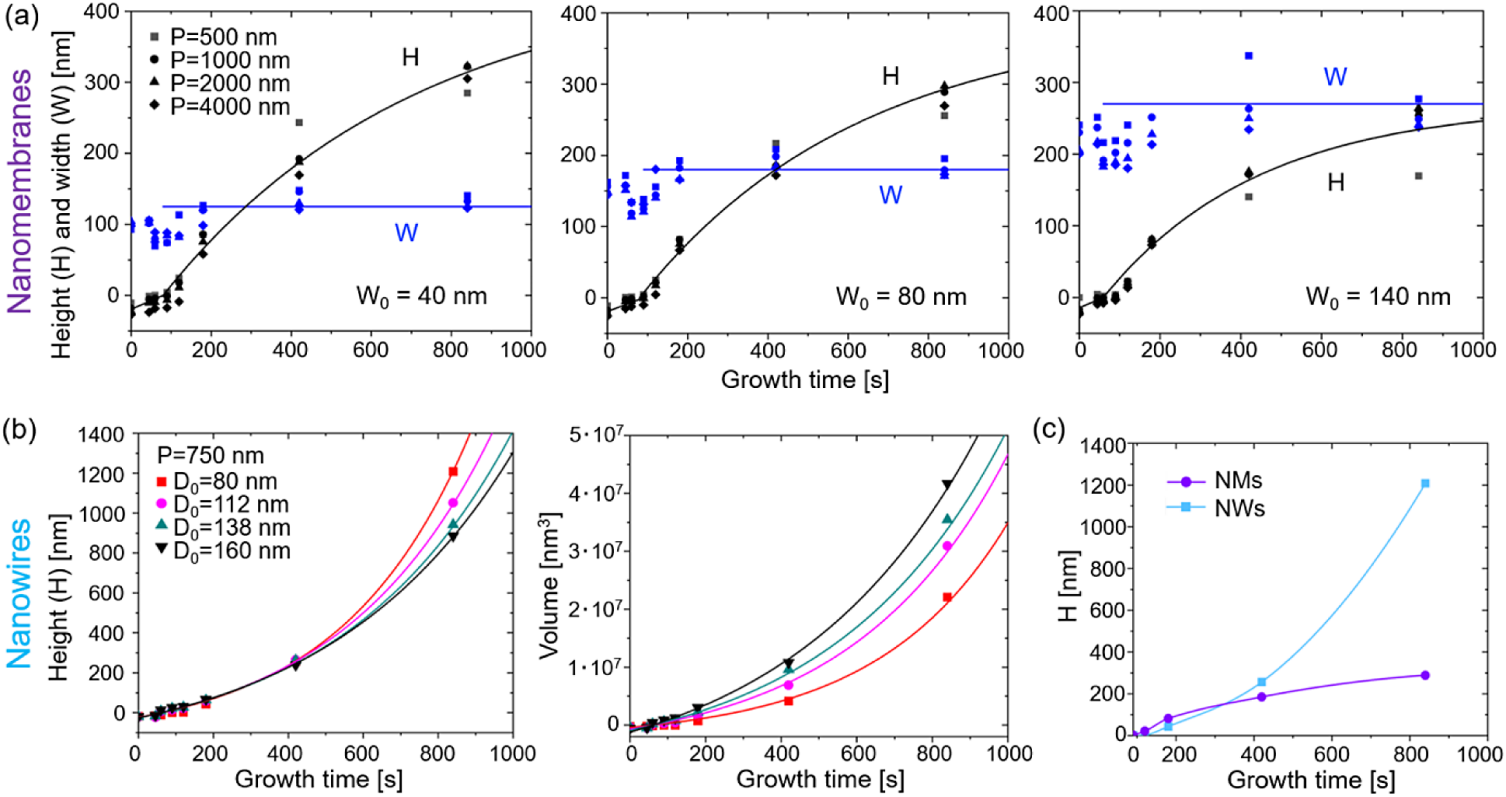
Growth kinetics of GaAs NMs and NWs. (a) Experimental data (symbols) and fits within the model (lines) for the time dependences of height *H* and width *W* of the NMs grown in arrays of the different pitches *P* shown in the legend and the three different nominal widths *W*_0_ = 40, 80, and 140 nm. (b) Height and volume of NWs grown in holes of different nominal diameters *D*_0_ shown in the legend. (c) Superlinear for NWs and sublinear for NM evolution of height with time for similar nominal size (*W*_0_ = 80 nm for NMs, *D*_0_ = 80 nm for NWs). In all graphs, zero height corresponds to the mask level.

We now consider the growth kinetics of NMs and NWs outside of the mask. The symbols in Figure 3a show the time evolution of the NM widths (*W*) and heights (*H*) for different nominal widths (*W*_0_) and pitches (*P*) of the slits. The procedure for the estimation of these parameters can be found in the Supporting Information. The symbols in Figures 3b show the time evolution of the NW heights and volumes for different nominal diameters of the openings at a fixed pitch of 750 nm. The solid lines represent the fits from the model, which will be discussed in the next section. The actual widths of NMs increase slightly at the beginning, corresponding to lateral growth outside the openings, but saturate to a constant value after about ∼200 s. A similar trend is observed for NWs, as demonstrated in Figure S6. Then, both NWs and NMs only grow vertically. No significant pitch dependence of the growth kinetics is observed for NMs, consistent with ref^59^. Therefore, surface diffusion of Ga adatoms from the mask surface^60^ or re-emission of Ga^65^ has almost no influence on the morphological evolution of NMs growing from a pitch-independent flux of Ga atoms per unit area. The lack of pitch dependency also indicates a nonsignificant contribution of diffusion of adatoms on the growth mask. After saturation of the widths and diameters, the volumes of the NM and NW increase as a result of their increasing heights. Thin NMs and NWs are systematically taller than their wider counterparts, which clearly indicates the diffusion-induced character of growth.^22,39−41,51,52,62^ However, the time evolution of heights is strikingly different, as demonstrated in Figure 3c, for the two types of structures with similar nominal dimensions of the openings. The evolution of the NW heights in Figure 3b is superlinear, meaning that higher NWs collect more Ga adatoms, contributing to their axial growth. This behavior is fully consistent with the previous works on vertical III–V NWs grown by the VLS or SAE methods in both MBE and MOVPE.^21,26,37,38,41,50,66^ As discussed in detail below, the superlinear growth kinetics of NWs are perfectly fitted by the exponential curves, which correspond to the collection of Ga adatoms from the entire length of NW sidewalls in the absence of radial growth.^22,36−41^ Conversely, the time evolution of the NM heights in Figure 3a is markedly sublinear for any width and pitch of the slit array. When we compare the growth of NMs having a nominal width equal to a nominal diameter of NWs, the time evolution of heights is clearly different, as demonstrated in Figure 3c.

Thus, our NMs and NWs have strikingly different kinetics even if they are simultaneously grown on the same substrate. This contrast can be uniquely related to their nature and dimensionality. In a complex picture in which both nanostructures remain solely restricted by {110} family side facets and the (111)B facet on top, a unified, self-consistent kinetic model is needed to pinpoint the key factors determining these distinct growth regimes.

## Modeling

**Figure 4:**
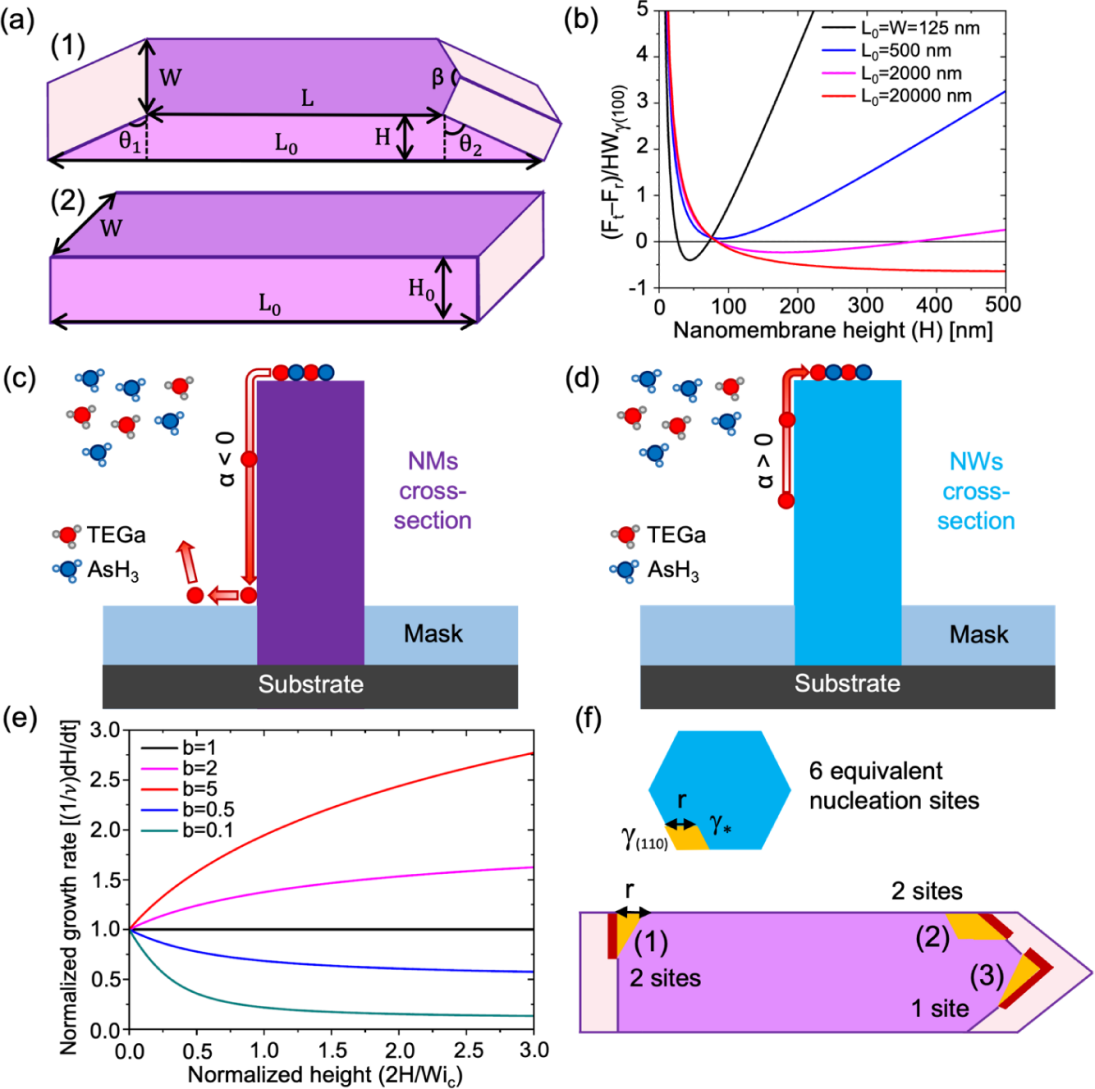
Self-regulation of the adatom diffusion flux. (a) Illustration of the NM geometries: the experimentally observed tapered shape versus rectangular shape with vertical sidewalls, two of which belong to the {211} family and have a higher surface energy than the (110) facets. The second geometry would correspond to lithographically patterned geometry protruding outside the mask. (b) Surface energy difference between the tapered and rectangular NMs of the same volume versus *H*, which becomes negative for long enough slits. (c, d) Illustration of negative diffusion flux of Ga adatoms on vertical (110) side facets of NMs and positive for NWs. (e) Evolution of the normalized growth rate as a function of the normalized height, obtained from eq 4 at different levels shown in the legend. The growth kinetics is linear at *b* = 1, sublinear at *b* < 1, and superlinear at *b* > 1. (f) Schematic of different nucleation sites in NWs and NMs in the case of corner nucleation. All 2D islands are restricted by the (110) facets. Red sides of islands nucleating on top of NMs indicate tapered facets.

In our model, we aim to understand the conversion of superlinear into sublinear growth in the transition from the symmetrical hexahedral geometry of NWs to the highly asymmetrical geometry of NMs elongated in the <11> direction and having tapered (110) side facets at both edges. We first describe the shape evolution of the NMs for the two geometries shown in Figure 4a. Figure 4a schematically represents an NM with tapered (111) edge facets (1) and a rectangular NM restricted by two long (110) facets and two short (211) facets at the edges (2), both at a given volume. The normalized difference of the surface energies of tapered (*F*_t_) and rectangular (*F*_r_) NMs having the same actual width of 125 nm and total volume is plotted in Figure 4b as a function of height *H* at different lengths of the slits *L*_0_ from 125 nm to 20 μm. The details of calculations using the surface energies of different planes of ZB GaAs γ_(111)B_, γ_(110)_, and γ_(211)_ and tapering angles θ_1_, θ_2_, and β are given in the Supporting Information. According to Figure 4b, the experimentally observed shape is preferred on surface energetic grounds for long enough NMs. In brief, the substitution of higher energy vertical (211) facets, inherited from the rectangular slit, with longer, but lower energy tapered (110) facets at the edges of the slits becomes interesting after *H* ∼ 50 nm at large enough *L*_0_. At these conditions, the energetically unfavorable elongation of the edge facets, formation of the ridge between them, and possible extension of the ridge onto the mask surface become unimportant in the total surface energy balance.

Modeling of the Ga-limited^50−52,60,62^ diffusion-induced growth of NMs and NWs in the VS mechanism is based on the transport-limited vertical growth rate:
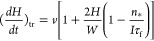
1

which has the same form for sufficiently long NMs and NWs if we replace the NM width *W* to the effective radius of hexahedral NW *R* = *W*/2 (see the Supporting Information for further details). Here, *I* is the vapor flux of Ga in nm^–2^ s^–1^, τ_f_ is the effective lifetime of Ga adatoms on the (110) sidewalls, n_*_ is the surface concentration of Ga adatoms on the top (111)B facet, and *v* = Ω*I* is the Ga deposition rate in nm/s, with Ω as the elementary volume per GaAs pair in the solid. The derivation of eq 1 is given in the Supporting Information and is not different from the standard model of NW growth.^22,37,39−42^ It assumes zero diffusion flux from the mask surface and the absence of Ga desorption from the NM/NW sidewalls, as suggested by the pitch-independent data reported in Figure 3. It also assumes that Ga adatoms do not evaporate from the NW/NM sidewalls, meaning that the maximum height of our structures (1200 nm for NWs) is smaller than the desorption-limited Ga diffusion length (∼1600 nm for SAE GaAs NWs grown by MOVPE at 750 °C according to ref^50^). This property is essential for the superlinear evolution of the heights of GaAs NWs seen in Figure 3b, without any noticeable transition to the linear growth mode. The absence of dangling bonds on the {110} sidewalls prevents nucleation from happening, thus promoting lateral growth.^22,65^ In this regard, the width *W* in eq 1 is considered time-independent because our structures do not extend in the lateral direction after a short incubation stage. However, we note that the unknown value of n_*_ in this model was always considered as a parameter such that n_*_Iτ_f_, which corresponds to positive diffusion flux from the NW sidewalls to its top. This condition would lead to the exponential increase in NW height with time, consistent with Figure 3b. Nonetheless, our data suggest that n_*_ becomes larger than *I*τ_f_ for NMs. This redirects the diffusion flux of Ga adatoms from the NM top to its sidewalls with subsequent evaporation.

We now try to understand the cause for the change in the direction of the diffusion flux from positive for NWs to negative for NMs, as illustrated in Figure 4c,d. We consider the nucleation-limited vertical growth rate in the mononuclear mode, determined by the nucleation rate of 2D islands on the top facet *J* times the surface area *S* available for nucleation,^40,43−47,67^
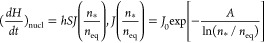
2This expression gives the nucleation probability on top of the structures per unit time, which equals the average nucleation-limited growth rate under the assumption of the instantaneous propagation of NW/NM monolayers. The nucleation rate depends on the adatom supersaturation φ_*_ = n_*_/*n*_eq_, with *n*_eq_ as the equilibrium concentration of Ga adatoms. The value of *J* is extremely sensitive to supersaturation because the surface energy constant, *A*, in the nucleation barrier under the exponent in eq 2 is on the order of 100 (the corresponding estimates are given below). The pre-exponent *J*_0_ depends weakly on supersaturation. This allows us to expand the nucleation barrier around the known supersaturation of vapor φ = *I*τ_f_/*n*_eq_,^40,43,67^
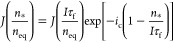
3with *i*_c_ = *A*/*I*τ_f_*/n*_eq_ ≫ 1 as the critical size (the number of GaAs pairs in the critical nucleus) of classical nucleation theory at supersaturation φ.^67^ Here, *J*(*I*τ_f_/*n*_eq_) is the nucleation rate at supersaturation φ, given by eq 2 at n_*_=Iτf.

Both transport-limited and nucleation-limited growth rates contain the unknown n_*_ in the difference (1 – n_*_)*/I*τ_f_, which determines the direction of Ga diffusion flux according to eq 1. To circumvent this uncertainty, we use the self-consistency condition (*dH*/*dt*)_tr_ = (*dH*/*dt*)_nucl_.^43,46,68^ Using eqs 1 and 3, we obtain the self-consistent vertical growth rate in the following form (see equation S5 for the detailed derivation):
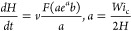
4

Here, *F*(*Z*) is a Lambert function, defined as an inverse function to the equation *F*_exp_(*F*) = *Z*. This growth law links the sign and magnitude of the adatom diffusion flux to parameter *b*, which equals the ratio of the nucleation-limited growth rate on top of NMs/NWs over the vapor flux of group III atoms *v*. By the definition of the Lambert function, we have *F*[*a*_exp_(*a*)] = *a.* This condition provides a connection between kinetic parameters so that they acquire an explainable physical meaning. Specifically, *dH*/*dt* = *v* at *b* = 1, i.e., the kinetic condition for a planar film. According to Figure 4e, *dH*/*dt* > *v* at *b* > 1 and *dH*/*dt* > *v* at *b* < 1. This result can be understood as follows: when *b* > 1, the nucleation-limited growth rate on the top facet at a given level of vapor supersaturation is higher than the vapor flux of Ga atoms. This difference is compensated by the positive diffusion flux of Ga adatoms from the NM/NW sidewalls to their tops. Conversely, at *b* > 1, the nucleation-limited growth rate is lower than the vapor flux. Therefore, the excessive Ga adatoms should be removed by negative surface diffusion from the NM/NW top to their sidewalls. Negative diffusion of group III adatoms was earlier discussed, for example, in ref^36^, for VLS GaAs NWs, but only in the absence of vapor flux of Ga. Self-regulation of the direction of the adatom diffusion flux by the nucleation rate on the top facet has never been considered, to our knowledge.

The general growth law given by eq 4 can be integrated only numerically. Our NWs and NMs are quite short, with heights smaller than 1200 nm for NWs and 300 nm for NMs. In this case, we can use eq 4 at *a*exp(*a*)*b* ≫ 1. Using the asymptote of the Lambert function at large *Z*, *F*(*Z*) ≅ ln *Z* – ln(ln *Z*), and *a* ≫ 1, we obtain a simplified growth law in the form
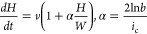
5

As in the general case, the direction of the adatom diffusion flux is determined by the value of *b*: the flux is positive at *b* > 1 and negative at *b* < 1. Integrating this with the initial condition *H*(*t* = *t*_0_) = 0, where *t*_0_ is the moment of time at which the structure reaches the height of the mask, we obtain
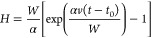
6

At α *>* 0, the height increases exponentially with time, as in our hexahedral NWs. At α > 0, the height evolution is sublinear, with a tendency to saturate in the large time interpolation, as in our long tapered NMs. The kinetic data for NMs and NWs in Figure 3a,b above the mask level are fitted by eq 6 with the parameters summarized in Table S1, where the width *W* is changed to the equivalent radius *R* for NWs. Growth inside the openings is fitted by linear curves. The fitting values of α are negative for all NMs, corresponding to sublinear growth kinetics, and decrease for larger *W*. The exponential growth of NWs is well-fitted using the same α value of 0.5 for NWs of different radii.

According to this analysis, the parameter *b* is larger than unity for NWs and smaller than unity for NMs. At the same supersaturation φ = *I*τ_f_*/n*_eq_ corresponding to the identical (110) side facets of NWs and NMs, the decrease of *b* for longer NMs can be due to (i) smaller nucleation area on top of NMs and (ii) larger surface energy constant *A* for islands nucleating on top of NMs, or a combination of these two factors. If the nucleation of 2D islands of identical shape (at *A* = const) were enabled on the entire top facets of NWs and NMs or along their perimeter, the nucleation rate would be the same for NWs and NMs. However, the nucleation area *S* is much larger for NMs, yielding a larger *b* for NMs than for NWs. This would lead to a higher growth rate and, hence, a larger height of NMs in comparison with NWs, which contradicts our experimental observations. Furthermore, the nucleation of the top of 20 μm long NMs would probably become polynuclear. This would further enhance the vertical growth rate of NMs, because polynuclear growth is faster than mononuclear.^43,44,67^ Therefore, we consider the nucleation scenarios shown in Figure 4f, where 2D islands nucleate at the corners of the NMs. This picture is similar to VLS GaAs NWs.^34^ In the SAE process, corner nucleation may be due to several reasons. First, the surface concentration of Ga adatoms may be locally higher at the corners. Second, surface passivation of inner facets by the excessive As atoms accumulated at the NM top may lead to γ_*_ > γ_(110)_, where γ_(110)_ is the surface energy of unpassivated (110) planes (see Figure 4f). Third, and most importantly, the tapered geometry of NMs can be preserved only when 2D islands nucleate with tapered facets, in which case the most probable nucleation site is the NM corner.^69^ Tapered facets are longer than vertical facets, which increases the surface energy constant *A* for NMs relative to NWs.

From eqs 2, 4, and 5, the parameter α can be presented in the form
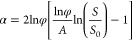
7

where *S*_0_ = *v*/[*hJ*_0_(φ)] ≅ const. This shows the importance of *A* in determining the sign of α. Considering the island shapes restricted solely to the {110} family facets with the maximum percentage of the outer facets, it can be shown that
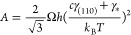
8

where *c* = 1 for trapezoid islands in the corners of hexahedral NWs and *c* > 1 for triangle or trapezoid islands in the corners of NMs due to a fraction of longer tapered facets. The detailed analysis is given in the Supporting Information. Specifically, for nucleation at position (1) in Figure 4f, we have , which equals 2 at θ_1_ = 54.3°. This yields *A*_NM_/*A*_NM_ = 2.17 at γ_(110)_ = 0.9γ_*_, which is a substantial difference. Using the parameters of ZB GaAs,^67,70^ Ω = 0.0452 nm^3^, *h* = 0.326 nm, and γ_(110)_ = 0.798 J/m^2^ at *T* = 800 °C, we obtain *A*_NW_*=* 214. For the fitting value of α_NW_ = 0.5 from Table S1, eq 7 gives ln(*S*_NW_/*S*_0_) = 70.4. For similar values of ln(*S*_NW_/*S*_0_) for NMs, eq 7 leads to the estimate α_NM_ ∼ −1, which corresponds to the negative fitting values for NMs given in Table S1.

From these considerations, the nucleation rate of monolayer islands on top of NMs results in many orders of magnitude lower than in NWs. The suppression of the nucleation rate originates from the tapered shape of islands nucleating on top of tapered NMs, in contrast to vertical NWs. This effect leads to *b* > 1 or α > 0 for NWs and *b* < 1 or α < 0 for NMs, which explains the observed transition from superlinear to sublinear growth. The growth of symmetrical GaAs NWs should become linear after their height reaches the diffusion length of Ga adatoms on the (110) facets.^37,38,40−42^ From the data, this diffusion length is larger than the maximum height of our NWs (∼1200 nm). Concerning elongated GaAs NMs, the current findings do not allow us to predict the conditions to achieve a linear growth regime.

## Conclusions

In conclusion, we quantitatively compared the morphology and growth kinetics of GaAs NWs and NMs grown in a single SAE process by MOVPE. The NWs have a symmetrical hexahedral shape, as broadly reported. We show that long enough NMs develop tapered (110) sidewalls rather than vertical {112} facets, consistent with previously reported MBE-grown ones. We demonstrate that this lateral tapering occurs for long enough nanostructures for surface energetic reasons. The observation of micrometer-sized monatomic terraces within the 1D slits clarifies that the growth of the NMs occurs layer-by-layer. As expected, the longitudinal propagation velocity appears to be the highest for nanostructures protruding from the mask. No pitch dependence of the shape and growth kinetics of NMs and NWs were observed under our MOVPE conditions. Both NMs and NWs do not grow laterally after a short incubation stage. Even though NWs and NMs are grown on the same substrate, the height evolutions of NMs and NWs appear strikingly different. The NW height follows the expected exponential dependence on time, while the NM height features a sublinear evolution. We explain this difference with a self-consistent model that links the direction and magnitude of the surface diffusion flux of Ga adatoms to the nucleation-limited growth rate on the top facet of the structures. The model provides a new expression for the growth rate of any structure formed by the direct impingement and surface diffusion of adatoms along its sidewalls. It gives a superlinear, linear, or sublinear height evolution when the nucleation-limited growth rate on the top facet is larger than, equal to, or smaller than the vapor flux of Ga atoms. We show that the nucleation rate of tapered islands on top of NMs is many orders of magnitude lower than that on top of vertical NWs, which explains the sublinear growth of NMs and superlinear growth of NWs at the same vapor supersaturation. We believe that the established self-regulation of the adatom diffusion flux by position- and shape-dependent nucleation on the top of the structures has far-reaching implications in different aspects of crystal growth and design. The findings presented herein hold significant implications for the morphological tuning of novel architectures in different materials systems and epitaxy techniques. These insights into growth mechanisms open up promising perspectives for the design of optoelectronic devices, from lasers to new-generation detectors.

## Methods

We use 2″ GaAs (111)B wafers where a SiO_2_ hard mask of 25 nm is deposited on the substrate by an Oxford Instrument Plasmalab System 100 and then patterned by electron beam lithography (Vistec EBPG5000ES). ZEP 20% diluted is the lithography resist. The pattern is transferred on the mask by an SPTS APS plasma etcher with a CHF_3_/SF_6_ mixture at an etching rate of 1 nm/s. After cleaning of resist residues with sonication in acetone and IPA, we smoothen the residual roughness by a wet etching step of 15 s in a buffered hydrofluoric acid solution, diluted 1:39 (in volume) in H_2_O. Samples are loaded into the MOVPE reactor, where they undergo an initial annealing step of 2 min at 850 °C under an AsH_3_ atmosphere. After annealing, the growths are carried out at a constant Ga growth rate, equivalent to 1.0 Å/s for planar growth, for different growth times to obtain nanostructures with different aspect ratios. Specifically, eight growth times are investigated: 30, 45, 60, 90, 120, 180, 420, and 840 s. Growths are performed at 800 °C at a V/III molar ratio equal to 1, whose corresponding mass fluxes are 10 sccm AsH_3_ and 50 sccm TEGa.

We characterize the morphology of NWs and NMs by SEM and AFM. Electron microscopy is performed with a ZEISS Merlin SEM at 3.00 kV and 100 pA. The topography of the nanostructures is investigated with a Bruker FastScan AFM in tapping mode with TESPA-V2 probes.

## Supporting Information Available

Additional experimental details and theoretical considerations, including position-dependent nucleation on top of long nanomembranes (PDF)
