# Supplementary material for: Nucleation-Limited Kinetics of GaAs Nanostructures Grown by Selective Area Epitaxy: Implications for Shape Engineering in Optoelectronics Devices
Source: ACS Appl Nano Mater. 2024 Aug 13;7(16):19065–74. doi: 10.1021/acsanm.4c02765 (PMC11348316; doi:10.1021/acsanm.4c02765)
Supplement: Supplementary file 1 — an4c02765_si_001.pdf [file an4c02765_si_001.pdf]

Supporting Information

Nucleation-Limited Kinetics of GaAs Nanostructures Grown by Selective Area Epitaxy: Implications for Shape Engineering in Optoelectronics Devices

Michele Zendrini[1], Vladimir Dubrovskii[2], Alok Rudra[1], Didem Dede[1], Anna Fontcuberta i Morral[1,3], Valerio Piazza[1*]

[1] Laboratory of Semiconductor Materials, Institute of Materials, Ecole Polytechnique Fédérale de Lausanne (EPFL), CH-1015 Lausanne, Switzerland
[2] Faculty of Physics, St. Petersburg State University, Universitetskaya Emb. 13B, 199034 St. Petersburg, Russia
[3] Institute of Physics, Ecole Polytechnique Fédérale de Lausanne (EPFL), CH-1015 Lausanne, Switzerland
Corresponding author : valerio.piazza@epfl.ch

Index

1. Annealing depths for NMs and NWs
2. Growth within the mask for NMs and NWs
3. Layer-by-layer growth in NMs
4. Nanomembrane shape
5. Transport-limited growth rates of NWs and NMs
6. Nucleation-limited growth rate and self-consistent growth equation
7. Position-dependent nucleation on top of long nanomembranes

1. Annealing depths for NMs and NWs

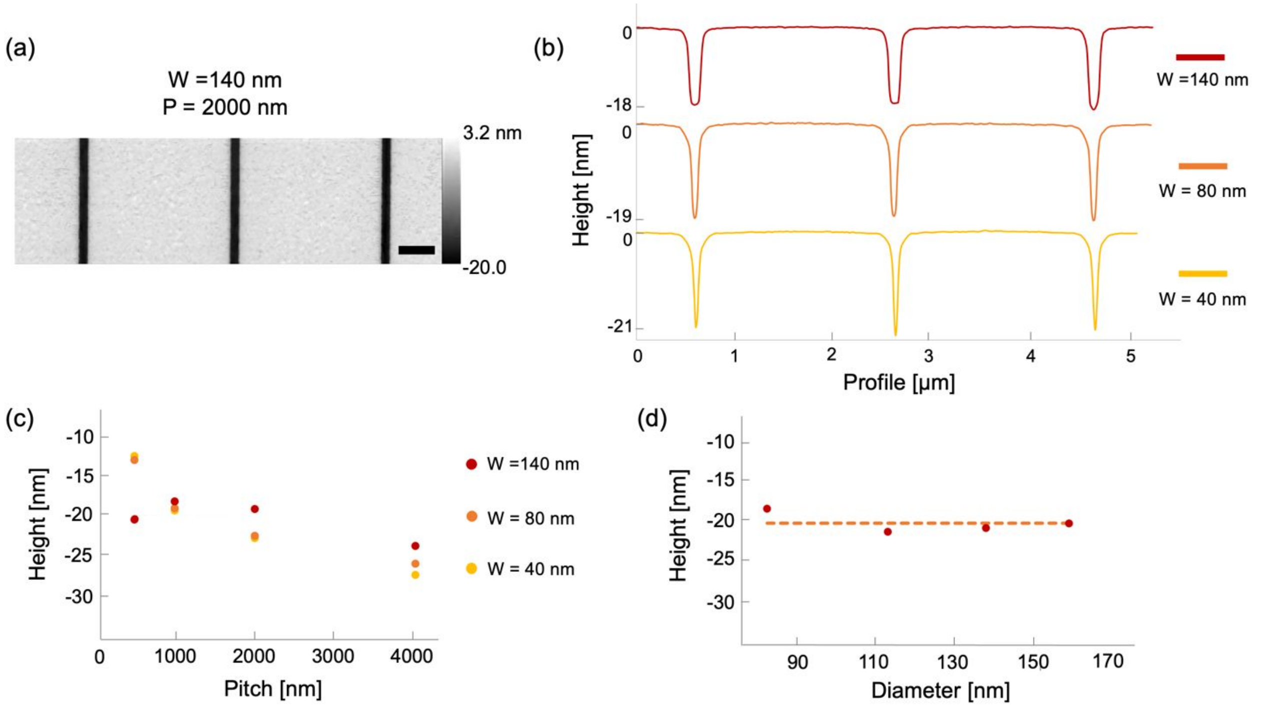

Figure S1. (a) AFM 2D map of annealed slits for NMs growth (W=140 nm, P=2000 nm, scale bar = 500 nm); (b) AFM line profiles of slits at different widths for a fixed pitch (P=2000 nm), showing reproducible homogeneity over the arrays in terms of depth; (c) Different annealing depths for NMs at different widths and pitches, showing variability due to atoms recollection; (d) Plot of the different annealing depths for NWs at different nominal diameters. Dashed line refers to average value.

After the annealing step, both slits and pinholes show reproducible depth in different structures over a single array, thus providing uniform initial condition for the growth. This homogeneity can be seen in Figure 1a-c for slits and figure 1d for holes. When considering different arrays, slits show more variability in depth rather than pinholes, as observable from the plot in Figure 1c. While holes with fixed pitch for all the diameters tend to have similar depths, slits show variability as a function of the pitch. This behavior can be explained considering atoms recollection from the material that is desorbed during annealing [1]. Slits at smaller pitches contribute with more desorption and hence can recollect more during the annealing, thus leading to smaller depths.

2. Growth within the mask for NMs and NWs

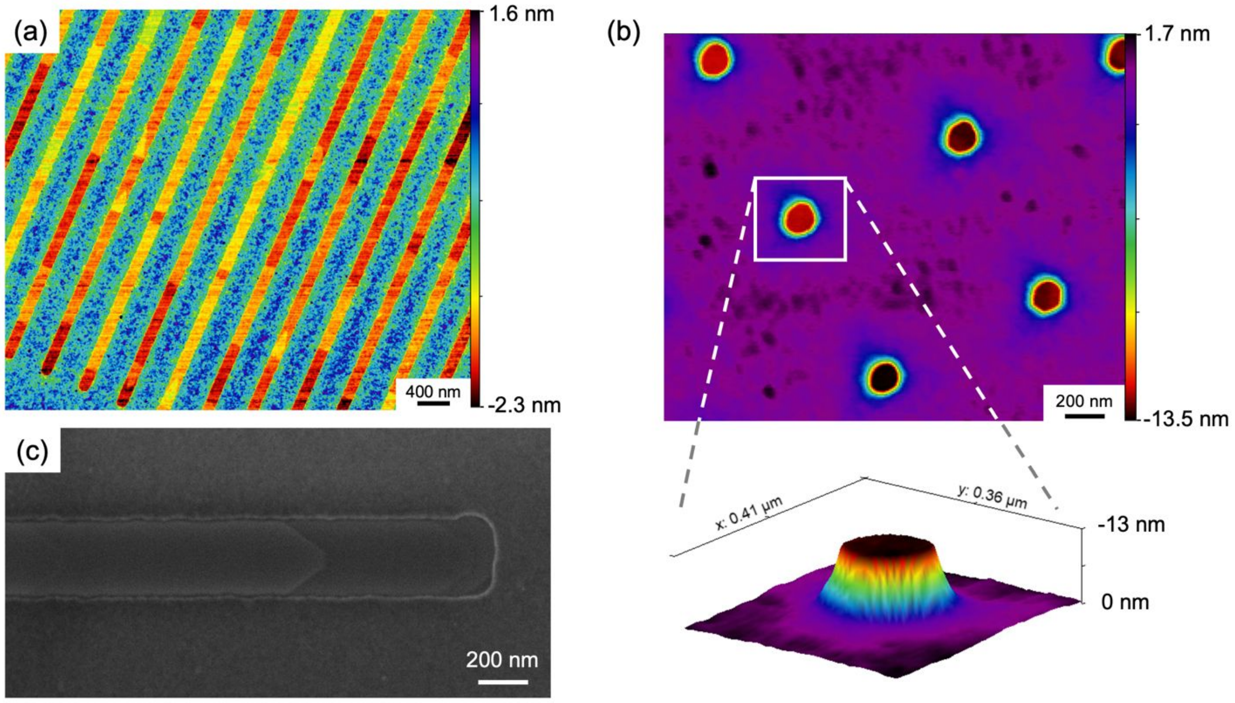

Figure S2. (a) AFM map of NMs (W=80 nm, P=500 nm) and (b) NWs (D=160 nm, P=750 nm) at 45 s of growth time. NMs show layer-by-layer growth within the mask with terraces and islands. NWs show filling of the pinholes at slightly different heights throughout the array and at a slower speed compared to NMs. (c) SEM image of NMs (W=140 nm, P=2000 nm) at 90 s of growth, showing the terraces at the end of the slits when reaching mask height.

Figure S2 (a) shows monolayer islands and terraces in the early stages of the NMs growth. Contrarily, the NWs do not show significant islands formation in early stages of growth within the mask, as it can be seen in Figure S2 (b). Such a difference can be reasonably due to the different exposed areas and filling volumes. Slits get filled more than holes for a fixed growth time. This trend is observed for all combinations of geometrical parameters that have been investigated.
The islands and terraces forming in the NMs tend to laterally expand with growth time until they are uniquely visible at the very end of the slits. Figure S2 (c) shows the presence of the terraces in the final part of the slits.

3. Layer-by-layer growth in NMs

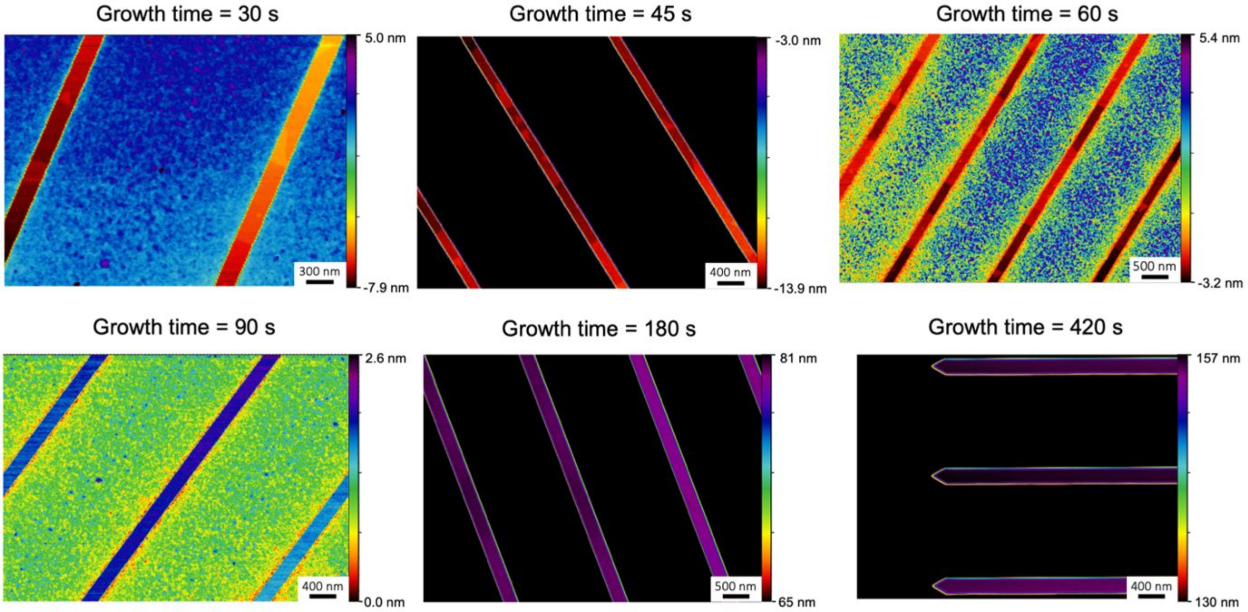

Figure S3. AFM 2D maps of NMs from the same array (w=140 nm, p=2000 nm) at different growth times, showing atomic terraces when NMs are still growing below the mask level and no atomic terraces when they protrude outside. The color legend has been rescaled at need to enhance the contrast on the growing top facet, thus maximizing the chance to observe atomic steps.

Figure S3 shows AFM scans of nanomembranes at different growth times. In the first growth stages when the nanostructures are still below the mask, islands and terraces of monolayer thickness are evident, showing layer-by-layer growth. Once nanostructures grow outside the mask, these terraces are no longer observable at any growth time.

4. Nanomembrane shape

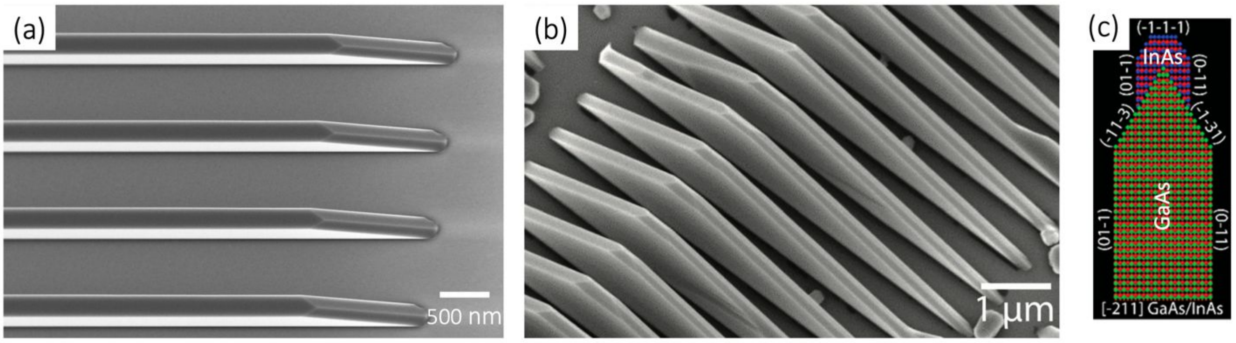

Figure S4. SEM tilted images comparing (a) NMs grown in this study (W=140 nm, P=1000 nm, t_{growth}= 840 s) with (b) the commonly described morphology from MBE-grown GaAs (111)B NMs[2]. In the panel (c), the modelled schematic of the growing facets for GaAs (111)B NMs used as templates for the growth of InAs nanowires is shown, highlighting the presence of (113) tapered top facets that are not present in our geometry [Reproduced from Ref. S3].

Our nanomembranes grow with flat (111)B top facet which is conserved throughout the growth in all the time window that has been investigated, as shown in Figure S4a. Such a morphology is markedly different with previously reported shapes[2,3] where the kinetics drive the appearance of (113) facets on top, leading to full completion of the structure into a triangular slab. Considering the saturation tendency of the sublinear growth of our NMs, such a morphology looks presumably far from being reachable.

As for the quantitative morphological description is concerned, the NMs are assumed to be infinitely long. The height (H) is obtained averaging over the AFM profile of different NMs belonging to the same array. The width (W) is obtained averaging over the values obtained from top-view SEM images of different NMs belonging to the same array. The volume per unit length (S) represents the vertical cross-section of the NMs and it is simply calculated by the product of their height and width.

To understand the shape of GaAs NMs grown in rectangular trenches of length L_0 elongated in <11-2> direction, we consider the tapered geometry shown in Figure S5 (a), where the NM has three inclined facets of lengths W, $W/[2\sin(\beta/2)]$ and $W/[2\sin(\beta/2)]$, where β is the angle of the ridge made by the two edge facets. The facet of length W makes the angle θ_1 to the vertical <111B> direction. The triangle edge is inclined at the angle θ_2 to the vertical. The volume V_t of a tapered NM having the base length L_0, top length $L = L_0 - H(tan\theta_1 + tan\theta_2)$, and height H above the mask level is given by:

$$V_t = WH\left[L_0 - \frac{H}{2}(tan\theta_1 + tan\theta_2)\right] + \frac{W^2}{4}cotan\left(\frac{\beta}{2}\right)\frac{H}{cos\theta_2}. \qquad \text{(S1)}$$

Here, the first term is the volume of trapezoid of width W, and the second term is the volume of triangle prism having the cross-sectional area $W^2 cotan(\beta/2)/4$ and length $H/cos\theta_2$. The volume V_r of rectangular NM of the same width W and length L_0 but different height H_0 (Figure S5b) restricted by two (110) and two (211) vertical side facets, equals:

$$V_r = WL_0H_0. \qquad \text{(S2)}$$

From Equations (1) and (2), the two NMs have the same volume ($V_t = V_r$) when:

$$H_0 = H\left(1 - \frac{H(tan\theta_1 + tan\theta_2)}{2L_0} + \frac{Wcotan(\beta/2)}{4L_0cos\theta_2}\right). \qquad \text{(S3)}$$

We now compare the surface energies F_t and F_r of the tapered and rectangular NMs shown in Figures S5a and S5b, respectively, under the condition of equal volumes. The experimentally observed NM shape should correspond to the lower surface energy[4]. The surface energy of tapered NM is given by:

$$F_t = W\left[L_0 - H(tan\theta_1 + tan\theta_2) + \frac{W^2}{4}cotan\left(\frac{\beta}{2}\right)\right]\gamma_{(111B)} +$$
$$\left[2HL_0 - H^2(tan\theta_1 + tan\theta_2) + WH\left(\frac{1}{cos\theta_1} + \frac{1}{\sin(\beta/2)cos\theta_2}\right)\right]\gamma_{(110)} + \frac{W^2}{4}cotan\left(\frac{\beta}{2}\right)\gamma_i.$$

(S4)

Here, the first term is the surface energy of the top (111B) facet, the second term is the surface energy of the (110) side facets, and the third term is the additional energy of the NM corner in contact with the mask surface, with the interfacial energy γ_i. The surface energy of rectangular NM equals:

$$F_r = WL_0\gamma_{(111B)} + 2H_0L_0\gamma_{(110)} + 2WH_0\gamma_{(211)}, \qquad \text{(S5)}$$

where the two vertical side (211) facets have the surface energy $\gamma_{(211)}$. Using Equation (3) for H_0 in Equation (5) and taking the difference $F_t - F_r$, the sign of $F_t - F_v$ at a given H is determined by:

$$\frac{F_t - F_r}{WH\gamma_{(110)}} = \frac{1}{cos\theta_1} + \left[\frac{1}{\sin(\beta/2)} - \frac{cotan(\beta/2)}{2}\right]\frac{1}{cos\theta_2} - (tan\theta_1 + tan\theta_2)\frac{\gamma_{(111B)}}{\gamma_{(110)}} +$$
$$\left(\frac{H(tan\theta_1 + tan\theta_2)}{L_0} - \frac{Wcotan(\beta/2)}{2L_0cos\theta_2} - 2\right)\frac{\gamma_{(211)}}{\gamma_{(110)}} + \frac{W}{4H}cotan\left(\frac{\beta}{2}\right)\frac{(\gamma_{(111B)} + \gamma_i)}{\gamma_{(110)}}.$$

(S6)

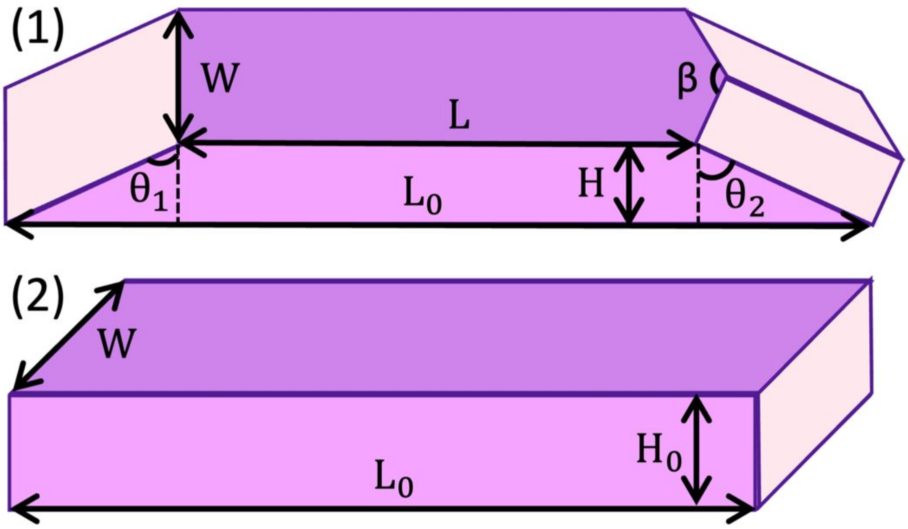

Figure S5. (a) Geometry of tapered NM having the width W, restricted by the two vertical side facets of the base length L_0 and height H, three inclined facets making the angle θ to the vertical, and the (111B) top facet parallel to the substrate surface. All side facets belong to the (111) family. (b) Rectangular NM of the same width W and length L_0 but different height H_0. The two side facets of rectangular NM belong to the (211) family, with a higher surface energy.

The angles ψ between the facets with the Miller indices $(h_1k_1l_1)$ and $(h_2k_2l_2)$ in cubic zincblende (ZB) lattice were obtained using:

$$cos\psi = \frac{h_1h_2+k_1k_2+l_1l_2}{\sqrt{h_1^2+k_1^2+l_1^2}\sqrt{h_2^2+k_2^2+l_2^2}}. \quad \text{(S7)}$$

This gives $\beta = 60^o$, $\theta_1 = 54.7^o$ (Ref. [5]), and $\theta_2 = 69.4^o$. The curves in Figure 4b, which is the same as in the main text, were obtained using the typical surface energy ratios of ZB GaAs planes (calculated, for example, in Refs.[S6-S8]): $\gamma_{(111B)}/\gamma_{(110)} = 0.865$, $\gamma_{(211)}/\gamma_{(110)} = 1.16$, assuming $\gamma_i/\gamma_{(110)} = 0.5$. The surface energy of the (211) family facets should be larger than that of the (110) family facets, which explains why hexahedral NWs grown in circular openings are restricted by six (110) side facets. On the other hand, tapered NMs have a smaller surface area of the lowest energy (111)B top facets. The surface energy of tapered NMs with three inclined facets, as in Figure S5a (the experimentally observed shape) is increased due to the additional surface and interfacial energy of the NM corner. This term is dominant for small heights H and suppresses tapering of such NMs in the initial growth stage. Further evolution of the NM shape with its height strongly depends on the length of rectangular trench L_0. According to Figure 4b, short NMs with $L_0 \sim W$ should become rectangular, while longer NMs with $L_0 >$ 2000 nm should taper after $H \sim 80$ nm and remain tapered for any height of interest. This explains the experimentally observed shape of NMs shown in Figure S5a on surface energetic grounds.

5. Transport-limited growth rates of NWs and NMs

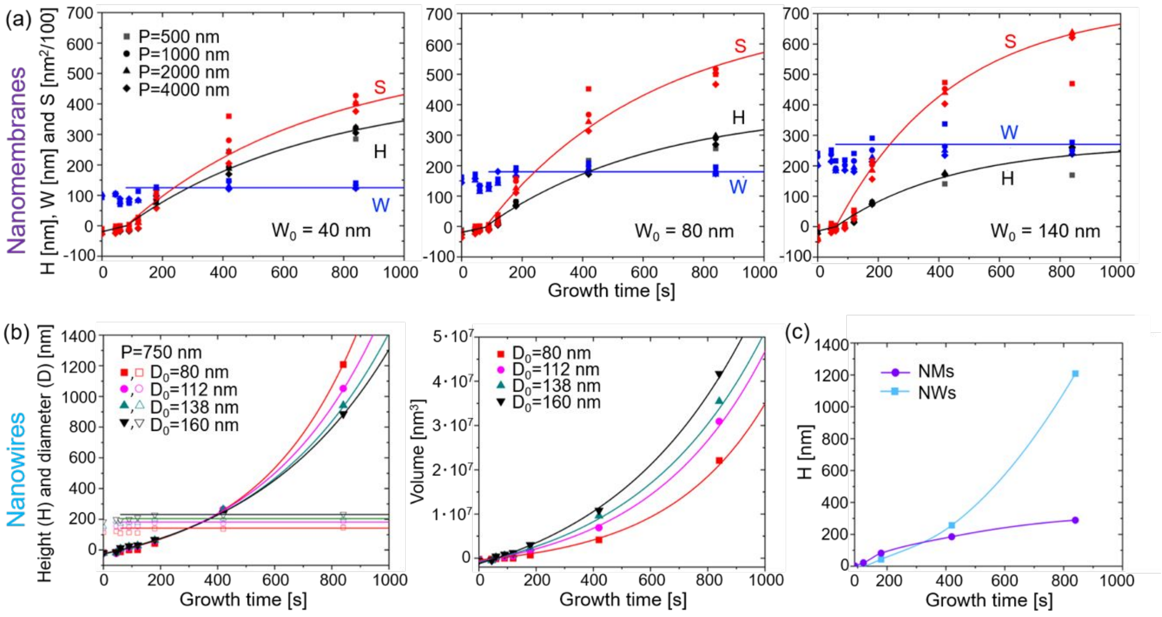

Figure S6. Growth kinetics of GaAs NMs and NWs. (a) Experimental data (symbols) and fits within the model (lines) for the time dependences of height H, width W, and volume per unit length S of the NMs grown in arrays of the different pitches P shown in the legend and the three different nominal widths W_0 = 40, 80 and 140 nm. (b) Height, diameter, and (c) volume of NWs grown in holes of different nominal diameters D_0 shown in the legend. (c) Superlinear for NWs and sublinear for NMs evolution of height with time for similar nominal size (W_0=80 nm for NMs, D_0=80 nm for NWs). In all graphs, zero height corresponds to the mask level.

Growth kinetics for NMs and NWs are fully described in Fig. S6. The symbols in Figure S6a show the time evolution of the NM widths (W) and heights (H), and volumes per unit length (S) for different nominal widths (W_0) and pitches (P) of the slits. The volume per unit length not only increases as the height, but also shows the evidence that bigger structures incorporate more material. In Fig. S6b, the symbols show the time evolution of diameters, heights and volumes of NWs at a fixed pitch of 750 nm. As no pitch-dependence has been observed for NMs, only one pitch has been considered for the analysis of NWs. The diameters follow the same trend as for NMs, where an initial increase reaches saturation after ~200 s.

We first consider the transport-limited axial growth rate of hexahedral NWs restricted by six equivalent vertical (110) facets of length $W/\sqrt{3}$ and height H. The growth is assumed to proceed under As-rich conditions, as usual in modeling the SAE process or VLS growth of III-V NWs[9]. The stationary diffusion equation for the surface concentration of Ga adatoms n_f on each sidewall facet is given by[S6]:

$$D_f \frac{d^2 n_f}{dz^2} + I - \frac{n_f}{\tau_f} = 0,$$

$$\left.\frac{dn_f}{dz}\right|_{z=0} = 0, \;\; n_f(z = H) = n_*. \qquad \text{(S8)}$$

Here, z is the vertical distance, D_f is the surface diffusion coefficient, τ_f is the characteristic desorption time and I is the flux of Ga atoms per unit area per unit time in $nm^{-2}s^{-1}$. The boundary condition at the NW base ($z = 0$) corresponds to zero diffusion flux from the mask surface. The second boundary condition at the NW top equates the concentration of sidewall adatoms at $z = H$ to the unknown adatom concentration on the top facet n_*. Solving Equation (S8), we calculate the diffusion flux of Ga adatoms per unit length per unit time:

$j = -D_f \frac{dn_f}{dz}\Big|_{z=H}$. (S9)

The result is given by:

$$j = I\lambda_f tanh\left(\frac{H}{\lambda_f}\right)\left(1 - \frac{n_*}{I\tau_f}\right), \quad \text{(S10)}$$

with $\lambda_f = \sqrt{D_f \tau_f}$ as the desorption-limited diffusion length of adatoms on the (110) NW sidewalls. It can be shown that the use of a more general boundary condition at the NW top of the Kramers type[9] modifies the value of the unknown n_*, but does not change the form of Equation (S10).

The diffusion-induced contribution to the axial growth rate of hexahedral NW having the volume $(\sqrt{3}/2)W^2H$ at a constant width W is obtained from:

$$\frac{1}{\Omega}\frac{\sqrt{3}}{2}W^2\left(\frac{dH}{dt}\right)_{diff} = \frac{6W}{\sqrt{3}}j, \quad \text{(S11)}$$

where Ω is the elementary volume per GaAs pair in solid. Inserting Equation (S10) into Equation (S11) leads to:

$$\left(\frac{dH}{dt}\right)_{diff} = v\frac{2\lambda_f}{R}tanh\left(\frac{H}{\lambda_f}\right)\left(1 - \frac{n_*}{I\tau_f}\right), \; R = \frac{W}{2}. \quad \text{(S12)}$$

Adding the direct impingement term:

$$\left(\frac{dH}{dt}\right)_{dir} = v \quad \text{(S13)}$$

to Equation (S12), the transport-limited growth rate of hexahedral NWs is obtained in the form:

$$\frac{1}{v}\left(\frac{dH}{dt}\right)_{tr} = 1 + \frac{2\lambda_f}{R}tanh\left(\frac{H}{\lambda_f}\right)\left(1 - \frac{n_*}{I\tau_f}\right), \quad \text{(S14)}$$

with R as the equivalent radius of hexahedral NW.

The transport-limited growth rate of long enough NMs in the geometry shown in Figure S5a is obtained as follows. The NMs have five facets, two of which are long and vertical, and the other three are short and inclined by the angles θ_1 and θ_2 to the vertical. Repeating the same calculations as above, the diffusion flux j_v originating from the long vertical facets is obtained in the form of Equation (S13):

$$j_v = I\lambda_f tanh\left(\frac{H}{\lambda_f}\right)\left(1 - \frac{n_*}{I\tau_f}\right). \quad \text{(S15)}$$

The diffusion fluxes j_{θ_1} and j_{θ_2} originating from the inclined facets with angles θ_1 and θ_2 to the vertical are given by:

$$j_{\theta_1} = I\lambda_f tanh\left(\frac{H}{\lambda_f cos\theta_1}\right)\left(1 - \frac{n_*}{I\tau_f}\right),$$
$$j_{\theta_2} = I\lambda_f tanh\left(\frac{H}{\lambda_f cos\theta_2}\right)\left(1 - \frac{n_*}{I\tau_f}\right), \quad \text{(S16)}$$

because the maximum distances along the inclined facets corresponding to the NW top equal $H/cos\theta_1$ and $H/cos\theta_2$. The total length of the two vertical facets at the NM top equals $2L = 2[L_0 - H(tan\theta_1 + tan\theta_2)]$. The length of the θ_1 facet equal W, and the total length of the two θ_2 facets equals $W/\sin(\beta/2)$. Differentiation of the NM volume given by Equation (S1) at $W = const$ equals the total diffusion current from the NM sidewalls to the top:

$$W\left[L_0 - H(tan\theta_1 + tan\theta_2) + \frac{W}{4}\frac{cotan(\beta/2)}{cos\theta_2}\right]\frac{1}{v}\left(\frac{dH}{dt}\right)_{diff} = \lambda_f\left(1 - \frac{n_*}{I\tau_f}\right)$$
$$\left[2(L_0 - H(tan\theta_1 + tan\theta_2))tanh\left(\frac{H}{\lambda_f}\right) + W tanh\left(\frac{H}{\lambda_f cos\theta_1}\right) + \frac{W}{\sin(\alpha/2)}tanh\left(\frac{H}{\lambda_f cos\theta_2}\right)\right].$$

(S17)

The total transport-limited vertical growth rate of NMs is obtained after adding the direct impingement term given by Equation (S13).
In the limit $H/\lambda_f \ll 1$, corresponding to the absence of desorption from the side facets, and for long enough NMs with $H/L_0 \ll 1$ and $W/L_0 \ll 1$, this yields the transport-limited growth rate of the form:

$$\frac{1}{v}\left(\frac{dH}{dt}\right)_{tr} = 1 + \frac{2H}{W}\left[1 + \frac{W}{2L_0}\left(\frac{1}{cos\theta_1} + \left(\frac{1}{sin\,(\beta/2)} - \frac{\text{cotan}\,(\beta/2)}{2}\right)\frac{1}{cos\theta_2}\right)\right]\left(1 - \frac{n_*}{I\tau_f}\right). \quad \text{(S18)}$$

At very large $L_0 = 20$ µm, as in our experiments, the edge terms in Equation (S17) can be safely neglected. This reduces Equation (S20) to the transport-limited vertical growth rate of infinitely long NMs (at $L_0 \rightarrow \infty$), considered in the main text:

$$\frac{1}{v}\left(\frac{dH}{dt}\right)_{tr} = 1 + \frac{2\lambda_f}{W} tanh\left(\frac{H}{\lambda_f}\right)\left(1 - \frac{n_*}{I\tau_f}\right). \quad \text{(S19)}$$

Therefore, the transport-limited growth rates of symmetrical NWs and infinitely long NMs, given by Equations (S14) and (S19) are identical if we replace the NM width W to the equivalent NW radius $R = W/2$. In particular, both equations are reduced to:

$$\frac{1}{v}\left(\frac{dH}{dt}\right)_{tr} = 1 + \frac{2H}{W}\left(1 - \frac{n_*}{I\tau_f}\right). \quad \text{(S20)}$$

in the absence of desorption from the NW/NM side facets.

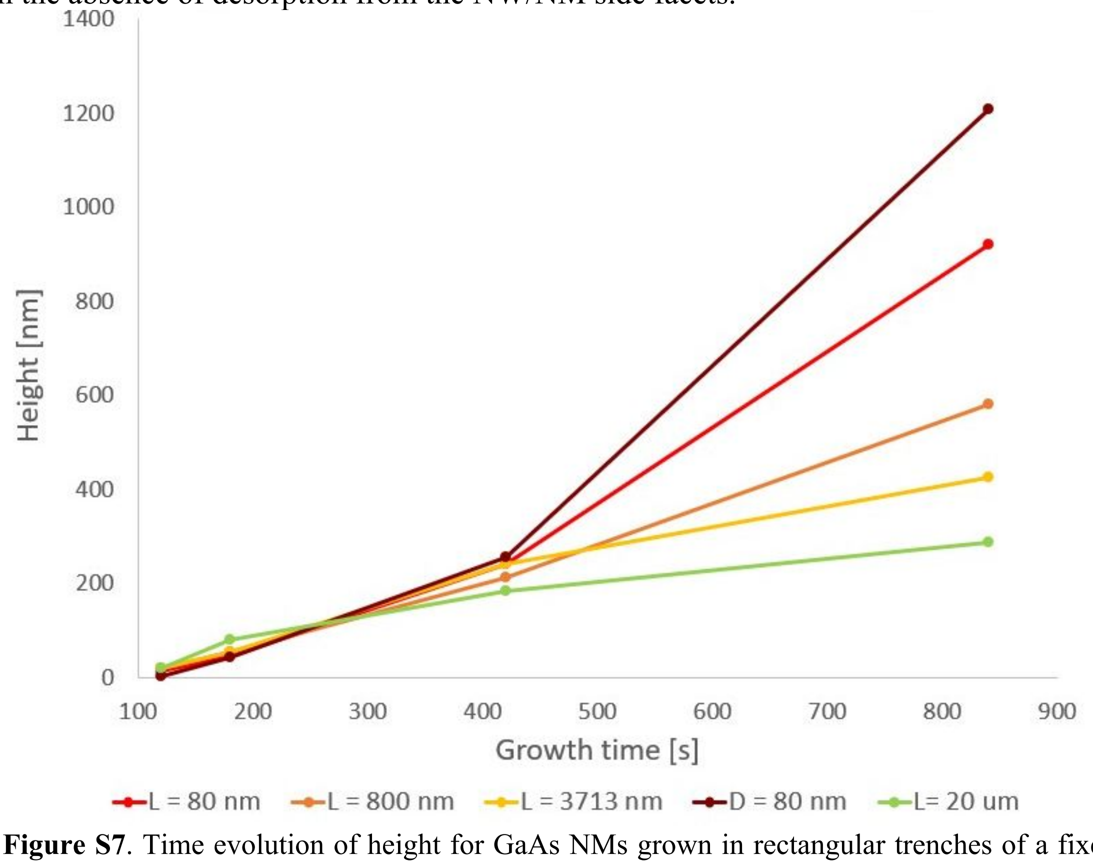

Figure S7. Time evolution of height for GaAs NMs grown in rectangular trenches of a fixed nominal width $W_0 =$ 80 nm and different lengths shown in the legend, compared to GaAs NWs grown in circular openings under the same conditions (the upper brown line). The transition from super-linear to sublinear behavior occurs for larger lengths.

The transport-limited growth rate of NMs given by Equation (S18) decreases with W/L_0, because the W/L_0 term in the bracket is positive. This corresponds to a decrease of the parameter b in the self-consistent growth model considered in the main text and hereinafter. The decrease of the growth rate for larger lengths is explained by the fact that the diffusion fluxes from the short edge facets of a NM become negligibly small in comparison with the

diffusion fluxes from the long sides at $W/L_0 \to 0$, but give a non-vanishing contribution for shorter NMs. This qualitatively explains the data shown in Figure S7, where the time evolution of the NM heights of a fixed width occurs for larger lengths L_0 or smaller aspect ratios W/L_0. For the sake of clarity, data in Figure S7 are qualitative and not conclusive, as the structures that are taken into consideration do not grow in regular arrays in the same manner as the NWs and NMs presented in this work.

6. Nucleation-limited growth rate and self-consistent growth equation

Most III-V NWs grow in the so-called mononuclear regime[9-15], in which the NW growth rate is determined by the waiting time between two successive nucleation events rather than by the lateral growth rate of two-dimensional (2D) island. In this mode, the monolayer propagation is almost instantaneous. We assume that the mononuclear growth regime occurs also in NMs after they grow out of the trenches. This assumption is supported by the fact that we never observe any steps on the top facets of such NMs, similarly to ZB III-V NWs grown by the vapor-liquid-solid method[5,16].

The nucleation-limited vertical growth rate of any structure in the mononuclear regime equals the nucleation rate J times the area S available for nucleation[9-12,14,15]:

$$\left(\frac{dH}{dt}\right)_{nucl} = hSJ\left(\frac{n_*}{n_{eq}}\right), \qquad \text{(S21)}$$

with h as the height of a monolayer. The nucleation rate depends on supersaturation on the top facet $\varphi_* = n_*/n_{eq}$, with n_{eq} as the equilibrium concentration of Ga adatoms. In classical nucleation theory, the nucleation rate is given by the Zeldovich formula:

$$J\left(\frac{n_*}{n_{eq}}\right) = J_0\left(\frac{n_*}{n_{eq}}\right)exp\left[-\frac{A}{\ln\left(n_*/n_{eq}\right)}\right], \qquad \text{(S22)}$$

which is valid for nucleation from liquid in the vapor-liquid-solid NWs[9-12,14] as well as for nucleation from 2D sea of adatoms in the vapor-solid growth[15]. The pre-exponential factor in this expression depends weakly on supersaturation. The exponential dependence is extremely steep, because the surface (or edge) energy constant A is much larger than unity. The value of A related to the surface energy of the island perimeter of monolayer height in thermal units, as discussed in detail below.

The transport-limited growth rate of NMs or NWs given by Equation (S19) contains the unknown n_*, which also enters the nucleation-limited growth rate given by Equation (S21). In the self-consistent approach[9,10,17], this uncertainty is circumvented using the condition that the two growth rates equal each other:

$$\left(\frac{dH}{dt}\right)_{tr} = \left(\frac{dH}{dt}\right)_{nucl}. \qquad \text{(S23)}$$

Using Equations (S19) and (S21), (S22), this is equivalent to

$$1 + \frac{2\lambda_f}{W}tanh\left(\frac{H}{\lambda_f}\right)\left(1 - \frac{n_*}{I\tau_f}\right) = \frac{hSJ_0\left(n_*/n_{eq}\right)exp\left[-\frac{A}{\ln\left(n_*/n_{eq}\right)}\right]}{v}, \qquad \text{(S24)}$$

with $W = R/2$ for hexahedral NWs. The steep exponential dependence of the Zeldovich nucleation rate on supersaturation allows us to use the asymptotic method of Refs.[9,10] for analytical solution of this equation for n_*. We approximate the nucleation barrier under the exponent in the right-hand side of Equation (S24) in the form:

$$\frac{A}{\ln\left(n_*/n_{eq}\right)} \cong \frac{A}{\ln\left(I\tau_f/n_{eq}\right)} + \frac{A}{ln^2\left(I\tau/n_{eq}\right)}\left(1 - \frac{n_*}{I\tau_f}\right), \qquad \text{(S25)}$$

and note that $A/ln^2\left(n_f/n_{eq}\right)$ equals the critical size (the number of GaAs pairs in the critical island) of classical nucleation theory at supersaturation $\varphi = I\tau_f/n_{eq}$:

$$i_c = \frac{A}{ln^2\left(I\tau_f/n_{eq}\right)}. \quad \text{(S26)}$$

Using Equation (S25) in Equation (S24), and introducing the new unknown:

$$x = i_c\left(1 - \frac{n_*}{I\tau_f}\right), \quad \text{(S27)}$$

Equation (S24) takes the form:

$$(a + x)e^x = b, \quad \text{(S28)}$$

as in the main text. The parameters are given by:

$$a = \frac{Wi_c}{2\lambda_f tanh\,(H/\lambda_f)}, \; b = \frac{hSJ\left(I\tau_f/n_{eq}\right)}{v}. \quad \text{(S29)}$$

The parameter b contains the nucleation rate at the known supersaturation $\varphi = I\tau_f/n_{eq}$, which is proportional to the vapor flux of group III atoms I. Clearly, this parameter equals the ratio of the nucleation-limited growth rate on top of NMs/NWs over the direct flux of group III atoms v. The NM/NW growth rate is given by

$$\frac{1}{v}\frac{dH}{dt} = 1 + \frac{x}{a}, \quad \text{(S30)}$$

where x is the solution to Equation (S28). This solution is expressed through the Lambert function $F(Z)$ such that $Fexp(F) = Z$:

$$x = F(ae^a b) - a. \quad \text{(S31)}$$

Substitution of this solution into Equation (S30) leads to the final result given in the main text:

$$\frac{1}{v}\frac{dH}{dt} = \frac{F(ae^a b)}{a}. \quad \text{(S32)}$$

By definition of the Lambert function, we have $F[aexp(a)] = 1$. Therefore, $dH/dt = v$ at $b = 1$. According to Figure 4e, which is the same as in the main text, $dH/dt > v$ at $b > 1$ and $dH/dt < v$ at $b < 1$. This result can be understood as follows. When $b > 1$, the nucleation-limited growth rate at a given level of vapor supersaturation is higher than the direct flux of Ga atoms. This difference is compensated by positive diffusion flux of Ga adatoms from the NM/NW sidewalls to their tops. Conversely, at $b < 1$ the nucleation-limited growth rate is lower than the direct flux. Therefore, the excessive Ga adatoms should be removed by negative surface diffusion from the NM/NW top to their sidewalls. Negative diffusion of group III adatoms was earlier discussed, for example, in Ref.[18] for VLS GaAs NWs. However, the dependence of the direction of the adatom diffusion flux on the nucleation rate on the top facet was never considered before to our knowledge.

Equation (S32) in the general case can be integrated only numerically. In this paper, we use the simplified growth law which follows from Equation (S32) at $aexp(a)b \gg 1$. At $H/\lambda_f \gg 1$, corresponding to the absence of desorption from the NM/NW sidewalls, the parameter a given by Equation (S29) becomes:

$$a = \frac{Wi_c}{2H}. \quad \text{(S33)}$$

Taking a plausible value of $i_c = 50$ and assuming $b > 0.1$, the value of $aexp(a)b$ is larger 3 for $W = 100$ nm and $H < 1000$ nm, while a is larger than 2.5. Using the known asymptotic behavior of the Lambert function at large Z, $F(Z) \cong lnZ - ln(lnZ)$, and $a \gg 1$, we obtain

$$\frac{F(ae^a b)}{a} \cong 1 + \frac{lnb}{a}. \quad \text{(S34)}$$

This reduces Equation (S32) to

$$\frac{dH}{dt} = v\left(1 + \alpha\frac{H}{W}\right), \; \alpha = \frac{2lnb}{i_c}, \quad \text{(S35)}$$

as in the main text. Figure S8 shows that this approximation is indeed accurate for large enough a.

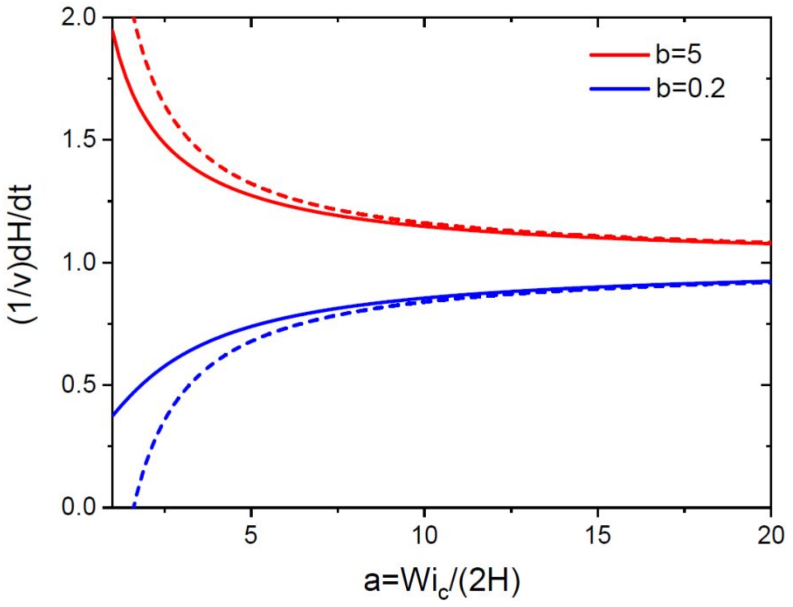

Figure S8. Normalized growth rates versus a, obtained from Equation (S32) for two different b shown in the legend (solid lines). Dashed lines show the approximation given by Eq. (S34).

Integrating Equation (S35) with the initial condition $H(t = t_0) = 0$, where t_0 is the moment of time at which the structure emerges above the mask level, we find:

$$H = \frac{W}{\alpha}\left[exp\left(\frac{\alpha v(t-t_0)}{W}\right) - 1\right]. \qquad \text{(S36)}$$

At $\alpha > 0$, the NW height increases exponentially with time, which is typical for short III-V NWs[9,18-20]. At $\alpha < 0$, the height evolution is sublinear with a tendency to saturation in the large time interpolation, as in our long NMs. As in the general case shown in Figure S6 the growth is super-linear at $b > 1$ and sublinear at $b < 1$. Equation (S36) is used in the main text for fitting the kinetic data for GaAs NMs and NWs with different α. The values of α are related to the parameters b according to Equation (S35).

The fitting parameters for Equation S36 are summarized in Table S1. The difference in the fitting values of $v = 0.7$ for NMs and $v = 0.41 - 0.47$ for NWs is not clear at the moment and requires further studies. It can be related to different coverages of the surface by the NWs and NMs, different local As/Ga ratios and other factors that are not taken into account in our Ga-limited growth model. Most importantly, the exponential growth kinetics of NWs is well-fitted with positive $\alpha = 0.5$, while fitting the sub-linear growth of NMs necessarily requires negative α.

Table S1: Fitting parameters for GaAs nanomembranes and nanowires in the simplified growth law of Equation S36

**Nanomembranes**				
Nominal width W_0 (nm)	$W = const$ (nm)	t_0 (s)	α	v (nm/s)
40	125	80	-0.278	0.71
80	180	80	-0.46	0.71
140	270	60	-1	0.7
**Nanowires**				
Nominal radius $R_0 = D_0/2$ (nm)	$R = const$ (nm)	t_0 (s)	α	v
40	70.9	60	0.5	0.41
56	90.9	60	0.5	0.458
69	102.3	60	0.5	0.45

80	115.7	60	0.5	0.466

7. Position-dependent nucleation on top of long nanomembranes

According to our analysis and fits to the kinetic data for MOVPE growth of long GaAs NMs versus symmetrical NWs given in the main text, the parameter b is larger than unity for NWs and smaller than unity for NMs. At the same supersaturation $\varphi = I\tau_f/n_{eq}$ corresponding to the identical (100) side facets of NWs and NMs, the decrease of b for longer NMs can be due (i) smaller nucleation area on top of NMs and (ii) larger surface energy constant A for islands nucleating on top of NMs, or a combination of these two factors. Indeed, from Equations (S29) and (S22) (at $n_* = I\tau_f$) we have:

$$b = \frac{S}{S_0} exp\left(-\frac{A}{ln\varphi}\right), S_0 = \frac{\Omega I}{hJ_0(\varphi)} \cong const. \qquad \text{(S37)}$$

Therefore:

$$b \propto S exp\left(-\frac{A}{ln\varphi}\right) \qquad \text{(S38)}$$

is primarily determined by S and A, and increases for larger S and smaller A.

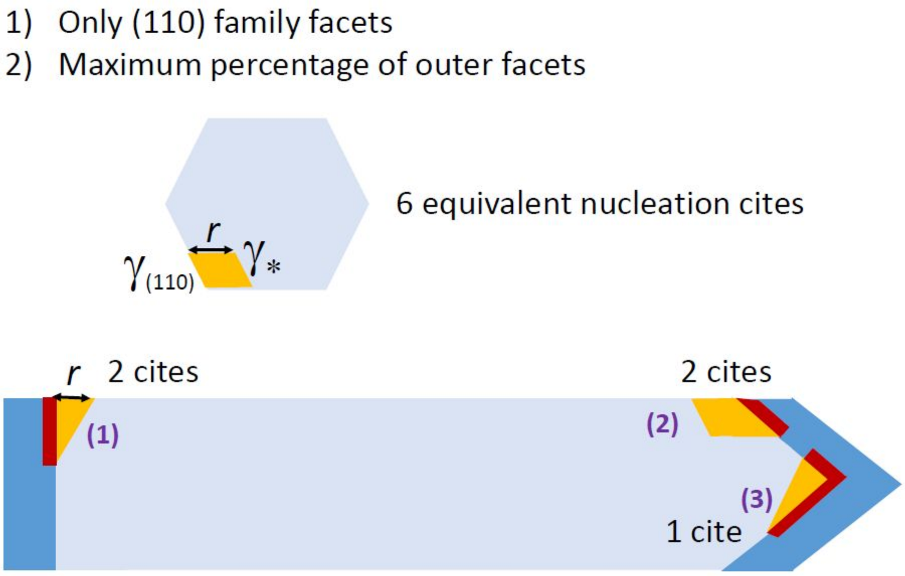

Figure S9. Illustration of the corner nucleation on top of symmetrical NWs and asymmetrical NMs. It is assumed that all islands nucleate with the (110) family facets and the maximum percentage of the outer facets. NWs have six equivalent nucleation cites for nucleation of trapezoid 2D islands of monolayer height h, restricted by vertical (110) facets. The surface energy of inner facets γ_* may be larger than $\gamma_{(110)}$ due to surface passivation by the excessive As atoms on the NW/NM top. NMs have five corners for nucleation of monolayer islands with (110) facets of shapes (1), (2) and (3). The tapered outer facets of these islands are shown in red. r is the linear size of the critical islands nucleated on top of NWs and NMs in position (1).

If nucleation of 2D islands of identical shape (at $A = const$) were enabled on the whole top facets of NWs and NMs or along their perimeter, the exponential factor in Equation (38) would be the same for NWs and NMs, while the nucleation area S would be much larger for long NMs. This would lead to a higher growth rate and hence larger height of NMs in comparison with NWs, which contradicts our experimental observations. Furthermore, nucleation of top of 20 µm long NMs would probably become poly-nuclear. This would further enhance the vertical growth rate of NMs, because poly-nuclear growth is faster than mononuclear[10,11]. We therefore consider the nucleation scenarios shown in Figure S9, where 2D islands nucleate at the corners of NMs. This picture is similar to VLS GaAs NWs[13]. In the SAE process, the corner nucleation may be due several reasons. First, the surface concentration of Ga adatoms may be higher at the corners.

Second, surface passivation of inner facets by the excessive As atoms accumulated at the NM top, may lead to $\gamma_* > \gamma_{(110)}$, where $\gamma_{(110)}$ is the surface energy of unpassivated (110) planes. Third, and most important, tapered geometry of NMs can be preserved only when 2D islands nucleate with tapered facets, in which case the most probable nucleation cite is the NM corner[16]. Tapered facets are longer than vertical, which increases the parameter A for NMs relative to NWs.
Considering the geometries of 2D islands restricted by the (110) facets with the lowest surface energy, and the maximum percentage of outer facets, as in Figure 4b, we note that their formation energy in thermal units of $k_B T$ can be presented in the form[15]:

$$F(i) = -\ln(\varphi)i + 2\sqrt{Ai} \quad \text{(S39)}$$

for any island shape, with a shape-dependent surface energy constant A. The first term corresponds to the energetically favorable decrease of chemical potential of i GaAs pairs in the vapor-solid phase transition driven by the supersaturation φ. The second term stands for the energetically unfavorable formation of the island surface. Maximizing this in i, the critical size and the nucleation barrier are obtained in the form $i_c = A/\ln^2\varphi$ and $F_c = F(i_c) = A/\ln\varphi$, as in Equations (S26), (S22). The number of GaAs pairs in the island is related to its surface energy s according to:

$$\Omega i = s(r)h, \quad \text{(S40)}$$

where r is a linear size of the island.
The shape of 2D island nucleating on top of symmetrical NWs is regular trapezoid with side r, restricted by four equivalent (110) facets. For nucleation at the corner of NMs in position (1) in Figure S9, the island shape is triangle with sides r, $\sqrt{3}r$ and $2r$, with the tapered side $\sqrt{3}r$ inclined by the angle θ_1 to the vertical direction. Assuming $r \gg h$, which is essential for use of macroscopic model for the island shapes, the surface area of the two islands is identical:

$$s(r) = \frac{\sqrt{3}}{2}r^2. \quad \text{(S41)}$$

However, the surface area of lateral facets of the NM island is noticeably larger due to tapering. Calculation of the surface energy terms for the NW and NM islands readily gives:

$$F_s^{NW}(i) = 2\frac{(\gamma_{(110)}+\gamma_*)}{k_B T}\left(\frac{2}{\sqrt{3}}\Omega h\right)^{1/2} i^{1/2}. \quad \text{(S42)}$$

$$F_s^{NM}(i) = 2\frac{(c\gamma_{(110)}+\gamma_*)}{k_B T}\left(\frac{2}{\sqrt{3}}\Omega h\right)^{1/2} i^{1/2},\ c = \frac{1}{2}\left(1 + \frac{\sqrt{3}}{\cos\theta_1}\right). \quad \text{(S43)}$$

Comparing these expressions with Equation (S39), we find the surface energy constants for NWs and NMs:

$$A_{NW} = \frac{2}{\sqrt{3}}\Omega h\left(\frac{\gamma_{(110)}+\gamma_*}{k_B T}\right)^2, \quad \text{(S44)}$$

$$A_{NM} = \frac{2}{\sqrt{3}}\Omega h\left(\frac{c\gamma_{(110)}+\gamma_*}{k_B T}\right)^2, \quad \text{(S45)}$$

The ratio of the two constants is given by:

$$\frac{A_{NM}}{A_{NW}} = \left(\frac{c\gamma_{(110)}+\gamma_*}{\gamma_{(110)}+\gamma_*}\right)^2. \quad \text{(S46)}$$

At $\theta_1 = 54.3^o$, the constant c in Equation (S43) equals 2, yielding A_{NM}/A_{NW}= 2.17 at $\gamma_{(110)} = 0.9\gamma_*$. Therefore, the surface energy constant for islands nucleating on top of NMs is more than two times larger than for NWs. It is easy to show that Equation (S46) holds for all the island shapes shown in Figure S9, where c is noticeably larger than unity. Consequently, the nucleation rate of monolayer islands on top of NMs is many orders of magnitude lower than in NWs. The suppression of the nucleation rate originates from the tapered shape of islands nucleating on top of tapered NMs, in contrast to vertical NWs. Using Equations (S37), (S35), the parameter α, which determines the direction of Ga diffusion flux, can be presented in the form:

$$\alpha = 2ln\varphi\left[\frac{ln\varphi}{A}ln\left(\frac{S}{S_0}\right) - 1\right]. \tag{S47}$$

Therefore, increasing the A value is responsible for changing the sign of α from positive in NWs to negative in NMs. Taking the parameters of cubic ZB GaAs[8,15]: $\Omega = 0.0452$ nm^3, $h = 0.326$ nm, $\gamma_{(110)} = 0.798$ J/m^2 at $T = 800$ ^{o}C, we obtain $A_{NW} = 214$. For the fitting value of $\alpha_{NW} = 0.5$, obtained from modeling of the super-linear growth kinetics of NWs, Equation (S46) gives $\ln\left(S_{NW}/S_0\right) = 70.4$. For similar values of $\ln\left(S_{NM}/S_0\right)$ for NMs, Equation (S47) leads to the estimate $\alpha_{NM} \sim -1$, which corresponds to the fitting values for NMs given in the main text.

In conclusion, we have explained the observed tapered shape of GaAs NMs grown in rectangular trenches elongated in the <11-2> direction. Tapering occurs due to the substitution of vertical (211) facets by the lower energy (110) facets, which becomes energetically preferred for large enough aspect ratios of the NMs. The diffusion-induced Ga-limited vertical growth rate of NWs and NMs has been considered in the self-consistent approach, where the unknown surface concentration of Ga adatoms at the top of the structures is related to the nucleation-limited growth rate in the mononuclear regime. We have shown that the nucleation rate on top of long tapered NMs is much lower than on top of symmetrical hexahedral NWs with vertical sidewalls. Insufficient nucleation rate of tapered islands on top of NMs redirects the diffusion flux of Ga adatoms from the NM top to their sidewalls, with subsequent evaporation. Consequently, the NM height evolves sub-linearly with time, in contrast to the exponential increase of the NW height. We believe that the established relationship between the direction of the diffusion flux and the position and shape-dependent nucleation rate on top of the structures has far-reaching implications in crystal growth in genera and will be useful for morphological tuning of a wide range of nanostructures in different material systems and epitaxy techniques.

References

[1] Morgan, Nicholas, et al. "From Layer-by-Layer Growth to Nanoridge Formation: Selective Area Epitaxy of GaAs by MOVPE." *Crystal Growth & Design* (2023).

[2] Tutuncuoglu, Gozde, et al. "Towards defect-free 1-D GaAs/AlGaAs heterostructures based on GaAs nanomembranes." *Nanoscale* 7.46 (2015): 19453-19460

[3] Friedl, Martin, et al. "Template-assisted scalable nanowire networks." *Nano Letters* 18.4 (2018): 2666-2671.

[4] Zeghouane, M.; André, Y.; Avit, G.; Jridi, J.; Bougerol, C.; Coulon, P.-M.; Ferret, P.; Castelluci, D.; Gil, E.; Shields, P.; Dubrovskii, V. G.; Trassoudaine. A. Formation of voids in selective area growth of InN nanorods in SiNx on GaN templates. *Nano Futures* **2020**, *4*, 025002.

[5)] Panciera, F.; Baraissov, Z.; Patriarche, G.; Dubrovskii, V. G.; Glas, F.; Travers, L.; Mirsaidov, U.; Harmand, J. C. Phase selection in self-catalyzed GaAs nanowires. *Nano Lett*ers **2020**, *20*, 1669.

[S6] Sibirev, N. V.; Timofeeva, M. A.; Bolshakov, A. D.; Nazarenko, M. V.; Dubrovskii, V. G. Surface energy and crystal structure of nanowhiskers of III–V semiconductor compounds. *Physics Solid State* **2010**, *52*, 1531.

[7] Rosini, M.; Magri, R. Surface effects on the atomic and electronic structure of unpassivated GaAs nanowires. *ACS Nano* **2010**, *4*, 6021.

[8] Pankoke, V.; Kratzer, P.; Sakong, S. Calculation of the diameter-dependent polytypism in GaAs nanowires from an atomic motif expansion of the formation energy. *Physical Review B* **2011**, *84*, 075455.

[9] Dubrovskii, V. G.; Hervieu, Yu. Yu. Diffusion-induced growth of nanowires: Generalized boundary conditions and self-consistent kinetic equation. *Journal of Crystal Growth* **2014**, *401*, 431.

[10] Dubrovskii, V. G.; Sibirev, N. V. Growth rate of a crystal facet of arbitrary size and the growth kinetics of vertical nanowires. *Physical Review E* **2004**, *70*, 031604.

[11] Kashchiev, D. Dependence of the growth rate of nanowires on the nanowire diameter. *Crystal Growth Design* **2006**, 6, 1154.

[12] Dubrovskii, V. G.; Sibirev, N. V.; Harmand, J. C.; Glas, F. Growth kinetics and crystal structure of semiconductor nanowires. *Physical Review B* **2008**, *78*, 235301.
[13] Harmand, J. C.; Patriarche, G.; Glas, F.; Panciera, F.; Florea, I.; Maurice, J.-L.; Travers, L.; Ollivier, Y. Atomic step flow on a nanofacet. *Physical Review Lett*ers **2018**, *121*, 166101.
[14] Glas, F.; Panciera, F.; Harmand, J. C. Statistics of nucleation and growth of single monolayers in nanowires: towards a deterministic regime. *Physica Status Solidi RRL* **2022**, *16*, 2100647.
[15] Dubrovskii, V. G. *Nucleation theory and growth of nanostructures*. Springer, Heidelberg – New York – Dordrecht – London, 2014.
[16] Wen, C.-Y.; Tersoff, J.; Hillerich, K.; Reuter, M. C.; Park, J. H.; Kodambaka, S.; Stach, E. A.; Ross, F. M. Periodically changing morphology of the growth interface in Si, Ge, and GaP nanowires. *Physical Review Lett*er **2011**, *107*, 025503.
[17] Glas, F.; Ramdani, M. R.; Patriarche, G.; Harmand, J. C. Predictive modeling of self-catalyzed III-V nanowire growth. *Physical Review B* **2013**, *88*, 195304.
[18] Dubrovskii, V. G.; Sibirev, N. V.; Cirlin, G. E.; Bouravleuv, A. D.; Samsonenko, Yu. B.; Dheeraj, D. L.; Zhou, H. L.; Sartel, C.; Harmand, J. C.; Patriarche, G.; Glas, F. Role of non-linear effects in nanowire growth and crystal phase. *Physical Review B* **2009**, *80*, 205305.
[19] Plante, M. C.; LaPierre, R. R. Analytical description of the metal-assisted growth of III–V nanowires: Axial and radial growths. *Journal of Applied Phys*ics **2009**, *105*, 114304.
[20] Harmand, J. C.; Glas, F.; Patriarche, G. Growth kinetics of a single $InP_{1-x}As_x$ nanowire. *Physical Review B* **2010**, *81*, 235436.
